# Differential response of human plasmacytoid pre-dendritic cells to SARS-CoV-2 variants

**DOI:** 10.1016/j.isci.2025.113394

**Published:** 2025-08-18

**Authors:** Daria Kartasheva-Ebertz, Dimitrios Topalis, Claudia Umana-Diaz, Okan Ayas, Laurine Couture, Pierre Tonnerre, Jasna Medvedovic, Laurent Meertens, Vassili Soumelis, Ali Amara

**Affiliations:** 1 Université Paris Cité, INSERM U976, Institut de Recherche Saint-Louis, Hôpital Saint-Louis, Paris, France; 2 Université Paris Cité, Biology of Emerging Viruses Team, INSERM U944/CNRS UMR 7212, Institut de Recherche Saint-Louis, Hôpital Saint Louis, Paris, France; 3 Assistance Publique-Hôpitaux de Paris, Hôpital Saint-Louis, Laboratoire d’Immunologie, Paris, France; 4 Université Paris-Cité, INSERM U976, Team ATIP-Avenir, Institut de Recherche Saint-Louis Paris, Paris, France

**Keywords:** Immune response, Virology

## Abstract

Severe acute respiratory syndrome coronavirus 2 (SARS-CoV-2) variants have been involved in various waves of the COVID-19 pandemic and showed different pathogenicity and inflammatory potential. Whether they can induce different patterns of innate immune activation in antigen-presenting cells is poorly understood. Here, we investigated the ability of primary plasmacytoid pre-dendritic cells (pDC), type 2 dendritic cells (DC2), and monocytes isolated from healthy donors to respond to SARS-CoV-2 variants. Transcriptomic profiling using RNA sequencing revealed that pDC respond differentially to SARS-CoV-2 variants, unlike DC2 and monocytes. Functional studies showed that pDC undergo differential activation programs upon SARS-CoV-2 variant stimulation. The Alpha and Delta variants induced P1-/P2-pDC effector phenotypes, characterized by strong IFN-α production. In contrast, the Omicron variant predominantly triggered a T cell-activating P3 phenotype, with lower IFN-α and IFN-λ production, and stronger proinflammatory and CD4^+^T cell responses. Our results indicate that SARS-CoV-2 variants can control pDC diversification pattern in different ways, which may influence disease severity.

## Highlights

• Plasmacytoid cells (pDCs) mount distinct immune responses to SARS-CoV-2 variants
• Omicron BA.1 induces P3-pDCs with reduced type I/III interferon transcripts
• P3-pDCs secrete more pro-inflammatory cytokines than P1-pDCs
• CD4^+^ T cell priming is stronger with Omicron BA.1 than Alpha or Delta variants

## Graphical abstract

**Figure:**
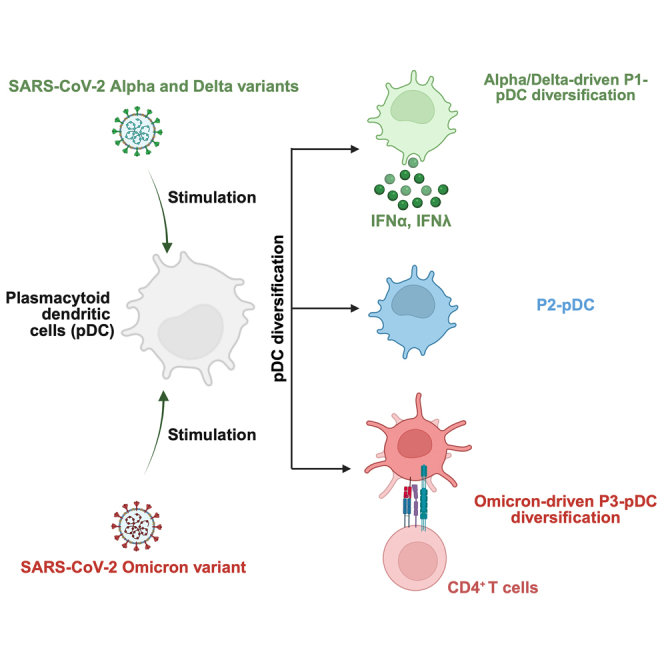

## Introduction

The COVID-19 pandemic affected more than 700 million and resulted in a dramatic amount of more than 7 million deaths worldwide since March 2020.^1^ This has led to a surge of research on the immune response to severe acute respiratory syndrome coronavirus 2 (SARS-CoV-2), the virus responsible for COVID-19. SARS-CoV-2 is a single positive-stranded RNA virus, belonging to betacoronavirus genera, with a lower mutational rate than in other RNA-containing viruses.^2^ However, it enables the virus to evolve relatively quickly into new viral variants that differ in terms of transmissibility, severity, immune response, and evasion.^3^ For now, there are five variants of concern (VOC): “Alpha”, “Beta”, “Gamma”, “Delta”, and “Omicron”.^3^ Pre-Omicron variants evolved independently from different lineages of the SARS-CoV-2 phylogenetic tree, whereas all Omicron subvariants stem from a common ancestral branch and diversify from there.^4^ Beside their evolutionary history, variants also differ in the rate of transmission as well as the severity of clinical disease.^5^ The highly transmissible Omicron (BA1 sub-lineage, R0 = 1.9) are less symptomatic and less likely to require hospitalization, predominantly infecting the upper respiratory tract predominantly unlike the Alpha and Delta variants.^6^ Nevertheless, little is known about the mechanistic differences in immune response between VOCs.

Antigen-presenting cells (APC) are the first line of anti-viral defense that intertwines innate and adaptive immune responses. After pathogen recognition, APC process the virus and enter into a maturation and differentiation program to trigger the appropriate and controlled innate and adaptive immune responses.^7^ Dendritic cells (DC) are professional APC that exist in various specialized subsets, including DC1, DC2, and DC3. Plasmacytoid pre-dendritic cells (pDC) and monocytes can further differentiate into DC following appropriate stimulation. A particular feature of pDC is that upon activation by a single stimulus, they can diversify into 3 different subsets defined by PD-L1 and CD80 expression levels: P1-pDC PD-L1^+^CD80^−^ produce large amounts of interferon alpha (IFN-α), P3-pDC PD-L1^−^CD80^+^ specialize in antigen presentation and T cell activation, P2-pDC PD-L1^+^CD80^+^ preform both functions.^8^

We were the first to demonstrate that the SARS-CoV-2 activates pDC in the absence of productive infection, via a pathway involving TLR7, UNC93B, IRAK4, and IRF7 pathway.^9^^,^^10^ Upon stimulation with the Alpha B220/95 SARS-CoV-2 variant*,* pDC diversify *in vitro* into P1, P2, P3 phenotypes, secrete large amounts of IFN-α, IFN-λ1, and proinflammatory cytokines, such as IL-6, IL-8, and IP-10^9^. Although type I and III IFN production by pDC during SARS-CoV-2 infection is associated with reduced viral replication via the induction of IFN-stimulated genes (ISGs),^11^^,^^12^ there is a complex relationship between IFN production, disease severity, patient age, anatomical site where IFN is secreted, and timing of IFN secretion.^13^ Several studies reported that severe-to-critical COVID-19 patients express high levels of IFN-α and IFN-λ2 in the upper and lower respiratory tract. Furthermore, these patients exhibit low ISG expression, accompanied by detrimental IFN effects in these compartments^14^^,^^15^ and a decrease in pDC numbers in the blood are observed in these patients.^16^^,^^17^^,^^18^ The timing of IFN-α and IFN-λ production plays a critical role in the beneficial or detrimental effects on patients.^19^^,^^20^

Given the known differences in the epidemiology and the clinical course of COVID-19 caused by SARS-CoV-2 variants, we investigated the hypothesis of variations in the APC response, particularly of pDCs, to these SARS-CoV-2 variants. Our study provides compelling evidence that pDC, unlike DC2 and monocytes, are exhibit distinct phenotypic and functional immune responses to SARS-CoV-2 variants *in vitro*.

## Results

### SARS-CoV-2 variants induce robust antiviral transcriptional programs in human pDCs

**Figure 1:**
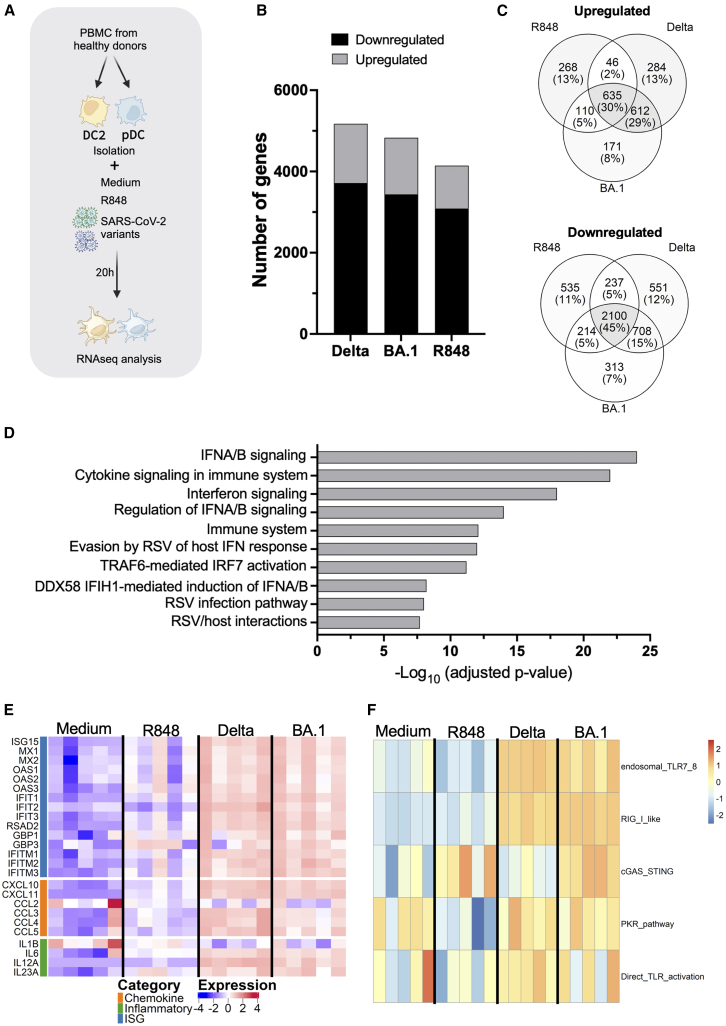
SARS-CoV-2 Delta and Omicron BA.1 variants induce robust gene expression changes in human pDCs RNAseq analysis of pDC purified population, stimulated for 20 h by Medium, R848, Delta or Omicron BA.1 SARS-CoV-2 variants. (A) Experimental workflow (Created with BioRender.com). (B) Stacked bar chart showing the number of differentially expressed genes (DEGs, adjusted *p* value <0.05) in pDCs after 20 h stimulation with Delta, Omicron BA.1, or R848 compared to unstimulated control. (C) Venn diagrams of the intersection of up and down differentially expressed genes across Delta, Omicron, and R848 treatments, revealing both common and stimulus-specific transcriptional signatures. (D) Pathway enrichment analysis of the core transcriptional signature shared by SARS-CoV-2 Delta and Omicron BA.1 variants in pDCs. (E) Expression heatmap of functionally categorized genes with robust upregulation of interferon-stimulated genes (ISGs), chemokines, and inflammatory mediators across Delta, Omicron BA.1, and R848 stimulations, highlighting the core antiviral response signature in pDCs. (F) Pathway enrichment analysis revealing virus-specific activation of multiple recognition pathways (endosomal and cytoplasmic sensors) compared to PKR and direct TLR7/8-restricted signaling by R848.

To identify different transcriptional and functional programs that may be induced by SARS-CoV-2 variants in human APC, we performed RNA sequencing (RNAseq) of pDC and DC2, isolated from 5 healthy donors, and stimulated with the Delta and Omicron BA.1 SARS-CoV-2 variants, respectively. Blood pDC and DC2 were sorted to reach at least 98% purity. Cells were cultured for 20 h in the presence of 100 RNA copies per cell of either SARS-CoV-2 Delta or Omicron BA.1 variant. In parallel medium alone or the TLR7/8 agonist resiquimod (R848) were used as a negative and positive control, respectively, for pDC activation (Figure 1A). First, we checked for subset-specific markers as control genes, across stimulation conditions. As expected, pDCs specifically expressed CD123, ILT7, and BDCA2, while DC2 expressed CD11C, DTX1, and PU.1, confirming cell type identity (Figures S1A and S1B).

RNA sequencing analysis revealed that both SARS-CoV-2 variants induced extensive transcriptional reprogramming compared to unstimulated controls (Figure 1B). Differential analysis revealed 5174 differentially expressed genes (DEGs) in pDC upon Delta stimulation (absolute fold change >2, FDR <0.05), including 1460 upregulated and 3714 downregulated genes, and 4830 DEGs upon Omicron BA.1 stimulation, including 1392 upregulated and 3438 downregulated genes. R848 stimulation resulted in approximately 4145 DEGs, indicating that viral stimulation triggered slightly more comprehensive transcriptional changes than the TLR7/8 agonist alone (detailed in individual volcano plots, Figures S1C–S1E). Notably, both viral variants preferentially induced downregulation of a series of genes, suggesting that recognition of SARS-CoV-2 might result in coordinated suppression of multiple cellular programs along with activation of antiviral pathways. Venn diagram analysis revealed substantial transcriptional overlap between stimuli, with 635 genes commonly upregulated across all three conditions, representing the shared antiviral response signature (Figure 1C, upper panel). Similarly, 2100 genes were commonly downregulated, indicating coordinated suppression of certain cellular programs during antiviral activation (Figure 1C, lower panel). Notably, most transcriptional changes were shared between variants. However, each stimulus also induced a distinct gene expression pattern: Delta uniquely upregulated 284 genes while Omicron BA.1 activated 171 variant-specific genes, suggesting potentially important differences in variant recognition and response mechanisms.

To characterize the core antiviral signature specifically shared by SARS-CoV-2 variants, we identified 612 genes commonly upregulated by both Delta and Omicron BA.1 variants (Figure 1D). This core viral signature represents the conserved transcriptional program activated regardless of variant-specific differences. Pathway enrichment analysis of this shared gene set (Figure 1D) demonstrated profound enrichment of interferon signaling pathways, with “interferon Alpha Beta signaling” showing the highest significance, followed by “cytokine signaling in immune system” and “interferon signaling.” The signature included 36 interferon-related genes, encompassing type I interferons (IFNA1-17, IFNB1), type III interferons (IFNL1-3), interferon-stimulated genes (ISG20, IFI35, IFI44L, IFIT2, IFIT5, and IFITM1-2), and key transcriptional regulators (IRF1, IRF9, and STAT2). These findings demonstrate that pDCs mount a highly conserved type I interferon-centered antiviral response across SARS-CoV-2 variants, indicating preserved innate immune recognition mechanisms.

Functional categorization of DEGs revealed robust activation of canonical antiviral pathways in pDCs following SARS-CoV-2 stimulation (Figure 1E). The interferon-stimulated gene (ISG) category showed the most pronounced upregulation across all viral stimuli, with key antiviral effectors including MX1, MX2, ISG15, ISG20, IFIT1, IFIT2, IFIT3, OAS1, OAS2, OAS3, and OASL displaying strong activation upon exposure to either Delta or Omicron BA.1 variants. These ISGs represent the core molecular machinery of the type I interferon response and demonstrate the potent antiviral state induced by SARS-CoV-2 recognition.^21^ Chemokine gene expression was similarly robust, with CXCL9, CXCL10, CXCL11, CCL2, CCL3, CCL4, and CCL5 markedly upregulated after viral stimulation, reflecting the recruitment and activation functions of pDCs during antiviral responses. The inflammatory category included genes such as IL-1β, IL-6, tumor necrosis factor α (TNF-α), and various inflammatory mediators that were coordinately activated, indicating a pro-inflammatory state accompanying the antiviral response. Importantly, the expression heatmap revealed that while R848 induced activation of ISGs and some inflammatory genes, the pattern and intensity differed from viral stimulation. SARS-CoV-2 variants induced a more comprehensive and intense activation of ISGs, particularly genes like IFIT family members, OAS genes, and MX genes, suggesting a more robust interferon pathway activation compared to an isolated TLR7/8 stimulation.

Pathway enrichment analysis revealed strong differences between viral recognition and synthetic TLR7/8 agonist R848 stimulation (Figure 1F). Both SARS-CoV-2 variants activated multiple pathways including “PKR_pathway”, “cGAS_STING”, “direct_TLR_activation”, “endosomal_TLR7_8”, and “RIG_I_like”, indicating engagement of diverse pattern recognition receptor systems. In contrast, R848 stimulation resulted in enrichment of the “PKR_pathway” with lower intensity, “direct_TLR_activation”, and “cGAS_STING”. This finding suggests that while R848 can activate “PKR-mediated signaling” and “direct_TLR_activation” pathways, it fails to engage the broader spectrum of recognition pathways activated by whole viral particles.

**Figure 2:**
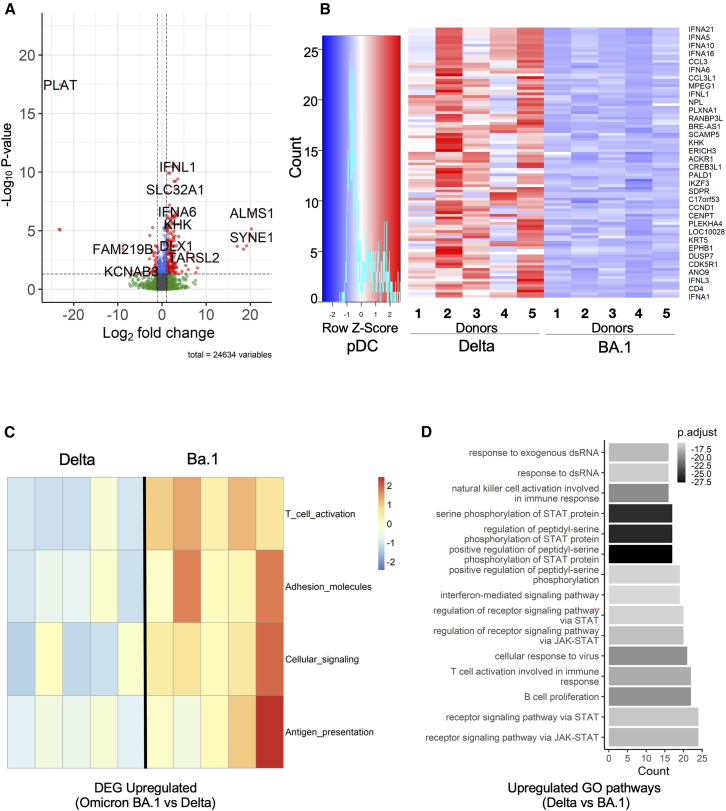
Delta and BA.1 SARS-CoV-2 variants elicit distinct immune response programs in pDC cells: enhanced interferon signaling by Delta versus improved antigen presentation by BA.1 (A) Differential gene expression analysis between Delta and Omicron BA.1 stimulated pDCs. (B) HeatMap of the first 100 DEGs in pDC stimulated by Delta vs. BA.1. (C) Functional annotation of upregulated genes in BA.1 vs. Delta stimulated pDC, showing T cell activation (B7-H3, KCNN4, BCL11B, XCR1, BATF, and ENTPD1), adhesion molecules (ITGA6, SPIRE1, FUT7, and TMEM255B), cellular signaling (RIN2, KCNN4, and MAPK12), and antigen presentation (EBF4, RIN2, LDLRAD3, and COLEC12). (D) Comparative analysis of enriched pathways in Delta-pDC vs. BA.1-pDC.

To compare transcriptional responses between Delta and Omicron BA.1 variants, we performed differential gene expression analysis on stimulated pDCs. The volcano plot revealed distinct expression patterns, with Delta stimulation driving upregulation of genes involved in cellular structure and mechanosensing (ALMS1, PIEZO2, SYNE1, and RAB3B) as well as interferon response genes (IFNL1, IFNA6, and IFNA8), while BA.1 stimulation preferentially induced coagulation-related genes, such as SERPINE1 and PLAT (Figure 2A). To further characterize these variant-specific responses, analysis of the top 100 DEGs upregulated in pDC stimulated with Delta as compared with Omicron BA.1 revealed distinct expression profiles (Figure 2B). Notably type I and III IFN genes were less induced under Omicron BA.1 stimulation in comparison with Delta. Conversely, functional annotation of genes upregulated in BA.1 versus Delta stimulation revealed enrichment in T cell activation pathways genes (B7-H3, KCNN4, BCL11B, XCR1, BATF, and ENTPD1), as well as adhesion molecules (ITGA6, SPIRE1, FUT7, and TMEM255B), cellular signaling components (RIN2, KCNN4, and MAPK12), and antigen presentation machinery (EBF4, RIN2, LDLRAD3, and COLEC12) (Figure 2C). Using pathway enrichment analysis, we identified pathways discriminating stimulation with Delta versus Omicron BA.1 (Figure 2D). We found that the “interferon-mediated signaling pathway”, “STAT protein regulation”, “JAK-STAT pathways”, “cellular response to virus”, “T cell activation involved in immune response”, and “B cell proliferation pathway” were upregulated under Delta stimulation (Figure 2D). These findings indicate that while BA.1 may be more efficient in promoting antigen presentation and T cell activation functions, the Delta variant triggers stronger type I interferon responses and downstream antiviral signaling cascades in pDCs.

Gene expression profiling of SARS-CoV-2-stimulated DC2 isolated from 5 healthy donors showed 3768 DEGs when compared with the non-stimulated control condition (absolute fold change>2, FDR <0.05), with 1634 were upregulated and 2134 downregulated genes. These included upregulated proinflammatory genes (IL-6, IL-2RA, CCL19, IL-13, IL-15), transcription factors (ZFPM2, EBF4, and ITGA1), enzymes (TNFAIP3), surface markers (CD80, CD302), Fc receptor (FCAMR) (Figure S1F). Unlike to pDCs stimulation, there was no distinct differential gene expression pattern between Delta and Omicron BA.1 stimulation in DC2 (Figure S1E). The upregulated GO pathways identified under SARS-CoV-2 (Delta and Omicron BA.1) stimulation compared to the non-stimulated control condition, included those associated with “CD4 T cell activation and differentiation”, “defense response to the virus”, “regulation of cell-cell adhesion”, “positive regulation of cytokine production”, and “regulation of viral genome replication”. This shows that the cells are in a functionally active state, ready to fulfill their antigen-presenting and antiviral roles (Figure S2A). “phagocytosis pathway”, as well as “negative regulation of cytokine production”, “leukocyte migration”, “ossification”, “taxis”, “small GTPase mediated signal transduction”, and “positive regulation of endocytosis” were less induced (Figure S2B). Overall, these data showed that SARS-CoV-2 variants Delta and Omicron BA.1 induced different transcriptional programs in primary pDC, but not in DC2.

### Omicron BA.1 variant skews human pDCs differentiation toward P3 subset

**Figure 3:**
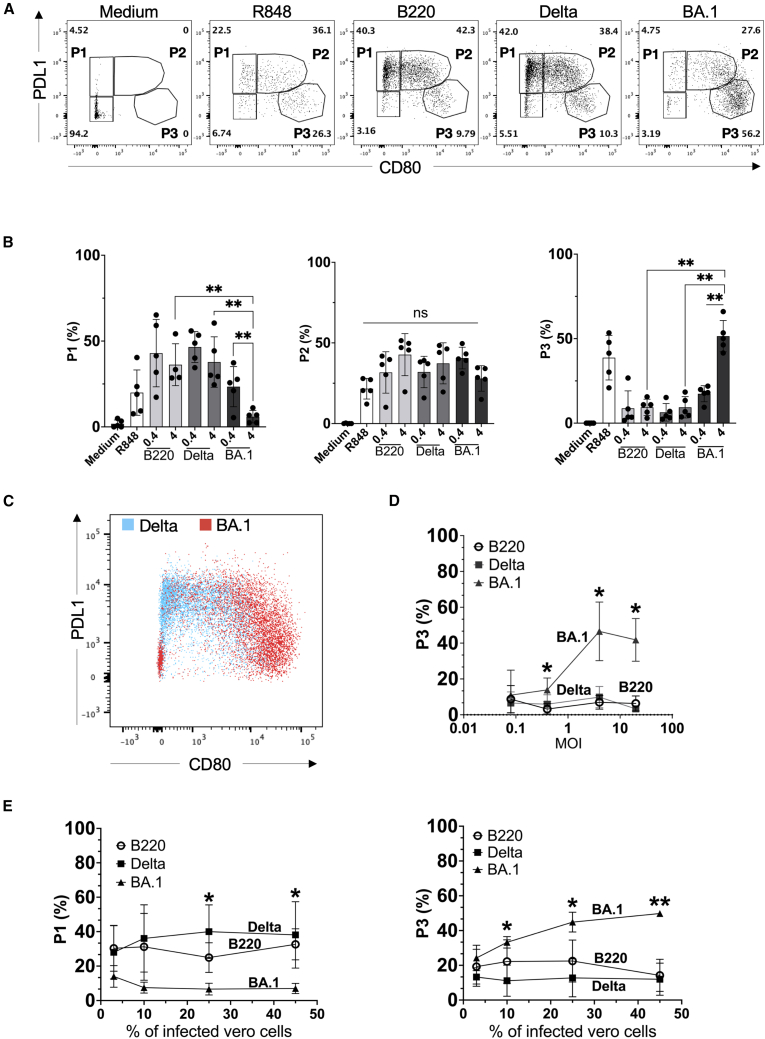
SARS-CoV-2 variant BA.1 shifts human pDCs differentiation to P3 phenotype. pDC activation by SARS-CoV-2 variants is dose-dependent and variant-dependent (A) Human pDC diversification, (defined by PDL1 and CD80 expression) upon Medium, R848 or SARS-CoV-2 variants (MOI = 4) stimulation. Representative plots from 1 healthy donor out of 5 is shown. (B) Quantification of P1, P2, P3 sub-populations in stimulated pDC. Five healthy donors from three independent experiment are shown. (C) Superposed dot plot of Unsupervised FlowSom analysis of concatenated samples of pDC+ Delta or BA.1 (MOI = 4, *n* = 8). (D) Human pDC were stimulated with SARS-CoV-2 variants at different concentrations (MOI = 0.08, 0.4, 4, and 12). The % of P3 pDC was assessed by flow cytometry based on PD-L1 and CD80 expression. Five healthy donors from three independent experiment are shown. (E**)** Same experiment than D except that pDC were co-cultured 20 h with Vero cells infected with SARS CoV-2 variants (3, 10, 25, and 45% of infected vero cells/pDC, P3, and P1 are presented). Four healthy donors are shown. Histograms represent means and bars SD. ∗, *p* < 0.05; ∗∗, *p* < 0.01; B— Mann-Whitney test; D, E two-way ANOVA test with Geisser-Greenhouse correction. Bars represent means ± SD.

The gene expression profiles obtained using RNA-seq suggested that the induction of type I and type III IFN genes is significantly weaker in Omicron BA.1-stimulated pDCs than in Delta-stimulated cells. This may indicate the existence of two different states of pDC in response to different variants of SARS-CoV-2, with distinct functional implications for the development of the immune response. To test those hypotheses, we investigated protein expression and functional states in primary human pDC. Previous studies indicated that a viral stimulus efficiently induces pDC diversification into IFN-producing cells and/or T cell-stimulating effectors.^8^ We assessed pDC stimulation in the presence of different SARS-CoV-2 viral variants: Alpha B220/95, Delta and Omicron BA.1. Human primary pDCs were purified from PBMCs of healthy donors, using a DC enrichment kit (Figure S3A) followed by cell sorting (Figure S3B) with 98% of purity (Figure S3C). After 20 h of the culture, all three variants induced pDC differentiation (Figure 3A). We observed a predominance of P3-pDC (PD-L1^−^CD80^+^) subset upon Omicron BA.1 and P1-pDC (PD-L1^+^CD80^−^) subset upon Alpha B220/95 or Delta stimulation (Figure 3B). Unsupervised FlowSom analysis (Figure 3C) was applied on concatenated samples of pDCs stimulated by Delta (*n* = 4) and Omicron BA.1 (*n* = 4) (Figure S3D). The analysis revealed two distribution patterns in line with manual gating: the P1- and P2-pDC subsets were predominant under Delta variant stimulation, while the P3- and P2-pDC subsets prevailed upon Omicron BA.1 stimulation.

To rule out the possibility that differences in pDC differentiation by SARS-CoV-2 variants depended on virus dose, we challenged human pDC with increasing concentrations of SARS-CoV-2 variants and assessed the percentage P3-pDC subset percentage. Omicron BA.1 variant induced sustained increase in the percentage of P3-pDC subset above 50%, while pDC stimulation with Delta and Alpha B220/95 variants already reached a plateau already at minimal viral dose without change thereafter, remaining at about 18% (Figure 3D). Similar results were obtained for P3-pDCs when pDC were co-cultured with Vero cells infected with different SARS-CoV-2 variants (Figure 3E). We also noted a sustained decrease of P1-pDCs under Omicron stimulation in Vero cells co-culture. In line with what was previously described in the literature,^9^ pDC and DC2 diversification by SARS-CoV-2 variants is independent of productive infection. Infection with Alpha 220/95, Delta or Omicron BA.1 SARS-CoV-2 variants is observed only in Vero cells, while in pDC and DC2 no SARS-CoV-2 N protein was observed. In addition, no viral RNA was detected in pDC when assessed by quantitative PCR after infection with variants Delta and Omicron BA.1 (Figures S4A and S4B).

We also assessed the outcome of Omicron BA.1 stimulation of DC2 (Figures S5A and S5B) and monocytes (Figures S5C and S5D) purified from the blood of healthy donors. Neither DC2 (Figure S6A) nor monocytes (Figure S6B) demonstrated differential activation upon stimulation with different SARS-CoV-2 variants, as evidenced by their consistent expression of PD-L1/CD80 (Figures S6C and S6D). However, Alpha B220/95 activated DC2 cells significantly less compared to Delta and Omicron BA.1 (Figures 6C and S7B). The DC2 activation was dose-dependent upon Omicron BA.1 (Figure S6C).

Together, these results showed that Omicron BA.1 drives pDC activation toward the P3-pDC subset, whereas Alpha B220/95 and Delta variants activate the P1-pDC subset. This difference in response to the variants was independent of the virus dose or the presence of productive infection. As neither DC2 nor monocytes showed a differential response, this response to SARS-CoV-2 variants is a special property of pDC.

### Stronger expression of co-stimulatory and migration markers in pDC following Omicron BA.1 exposure

**Figure 4:**
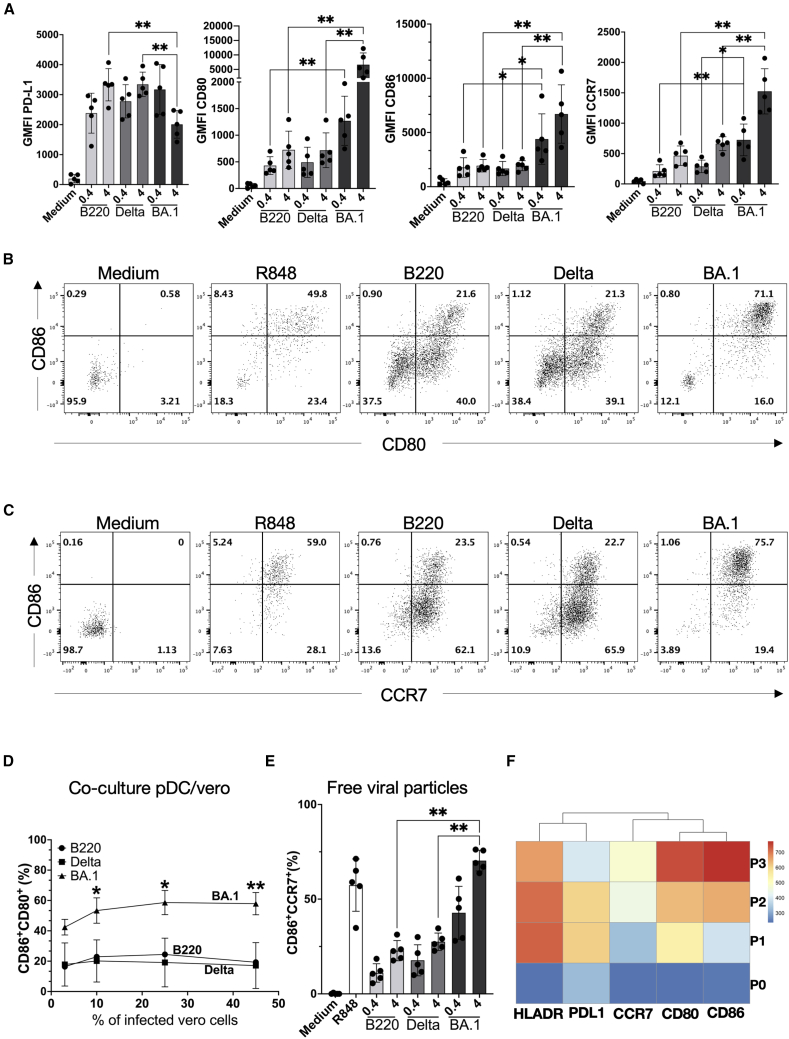
Stronger expression of co-stimulatory and migration markers in pDC following Omicron BA.1 exposure (A) Geometric mean of fluorescence intensity for PD-L1, CD80, CD86, and CCR7 surface markers on pDC stimulated by Medium, B220, Delta, or BA.1 with MOI = 0.4 or 4, for 20 h, *n* = 5. (B) Representative flow cytometry plots of CD80/CD86 expression on pDC upon Medium, R848 and SARS-CoV-2 variants stimulation. (C) % of CD86+CCR7+ cells upon SARS-CoV-2 variants stimulation**.** (D) % of CD80^+^/CD86^+^ pDC cells co-cultured with 3%, 10%, 25%, and 45% of infected Vero cells by SARS-CoV-2 variants. (E) % of CD86+CCR7+ cells upon pDC stimulated with SARS COV2 variants at two doses of virus. (F) Heatmap of HLA-DR, PD-L1, CCR7, CD80, and CD86 expression in pDC SARS-CoV-2 activates samples (unsupervised FlowSom analysis, *n* = 8). ∗, *p* < 0.05; ∗∗, *p* < 0.01; A, D— Mann-Whitney test; C— two-way ANOVA test with Geisser-Greenhouse correction. Bars represent means ± SD.

Co-stimulatory (CD80, CD86) and migratory (CCR7) markers play a key role in anti-viral immunity and serve as distinctive markers of a specific pDC phenotype that can activate naive T cells.^22^ Therefore, we questioned whether we would observe differences in the expression of these markers on pDC stimulated by Alpha B220/95, Delta and Omicron BA.1 SARS-CoV-2 variants. For this, human pDC were cultured for 20h in the presence of the SARS-CoV-2 variants. The expression of CD80, CD86, and CCR7 was assessed by flow cytometry. CD86, CD80, and CCR7 expression was significantly stronger (3- to 5-fold) upon Omicron BA.1 stimulation compared to Alpha B220/95 or Delta (Figure 4A). This was accompanied by a significantly higher percentage of CD80^+^CD86^+^ (Figures 4B and 4D) and CD86^+^CCR7^+^ (Figures 4C and 4E) double-positive pDC.

Almost 70% of Omicron BA.1-stimulated pDC were CD80^+^CD86^+^CCR7^+^ as compared with 20% of Alpha B220/95- or Delta-stimulated pDC. This phenotype corresponds to the aforementioned P3-pDC subset (Figure 4F), which migrates toward lymph nodes and primes naive T cells, as shown previously.^8^ No differences in activation markers expression were seen in DC2 (Figures S7A and S7B) or monocytes (Figures S7C and S7D) when stimulated with the three viral variants.

### Functional consequences of pDC activation by SARS-CoV-2 variants

**Figure 5:**
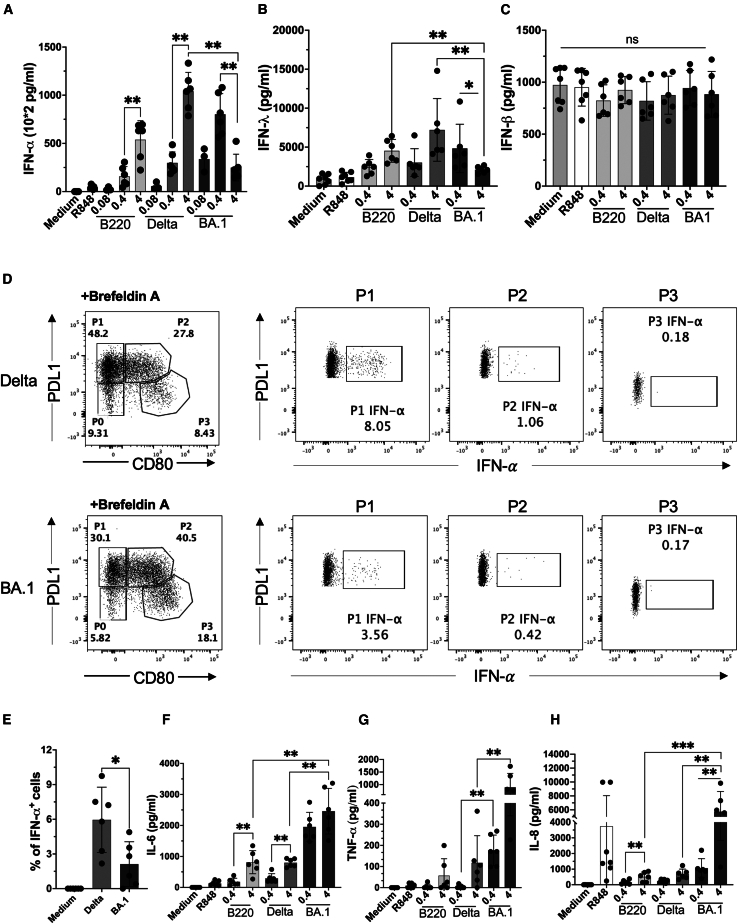
Functional consequences of pDC SARS-CoV-2 variant activation (A–D) P3 pDC phenotype under BA.1 stimulation have reduced secretion of IFN-**α** and IFN-**λ** and increased secretion of proinflammatory cytokines IL-6 and IL-8 in comparison with P1/P2 phenotype under B220 and Delta variants. (A) pDC IFN-**α**. (B) pDC IFN-**λ** (C). pDC IFN-*β* (D). Representative plots of pDC intracellular production of IFN-**α** after SARS-CoV-2 variant stimulation. (E) Percentage of pDC INF-**α**^+^ cells after 20 h SARS-CoV-2 viral variant stimulation. (F) pDC dose-dependent secretion of IL-6. (G) pDC dose-dependent secretion of TNF-**α**. (H) pDC dose-dependent secretion of IL-8. ∗, *p* < 0.05; ∗∗, *p* < 0.01; Mann-Whitney test. Bars represent the mean values ± SD of *n* = 6 healthy donors from three independent experiments.

A major component of antiviral defense is pDC-derived types I and III IFN.^12^ To understand the functional consequences of the established transcriptional and phenotypic differences in the pDC response to viral variants, we measured the production of key cytokines at the protein level following 20 h of stimulation. The transcriptomic signature revealed that (Figure 2B) Omicron BA.1-stimulated pDC displayed lower IFN-α and IFN-λ genes expression than Delta variant-stimulated cells. At the protein level, we observed a significant increase in IFN-α secretion upon Omicron BA.1 stimulation when increasing the MOI from 0.08 to 0.4, followed by a significant decrease of IFN-α concentration as viral load increased from MOI 0.4 to 4 (Figure 5A). In contrast, pDCs exposed to Alpha B220/95 and Delta displayed a peak of IFN-α secretion around 500 to 1000 ng/mL for the highest viral input (MOI 4), respectively. The same pattern was observed for IFN-λ with increasing secretion upon exposure to Alpha B220/95 and Delta variants and decreasing secretion when pDCs were stimulated with Omicron BA.1 (Figure 5B). Conversely, IFN-β secretion remained unaffected regardless of the viral variant used to challenge pDCs (Figure 5C).

Previously our group showed that IFN-α was secreted mainly by the P1-pDC subset,^8^ which may explain the reduced secretion upon Omicron BA.1 activation, where the P3 subset predominates. To confirm this, we performed IFN-α intracellular staining in purified pDC from healthy donors, after 20 h of culture with Delta or Omicron BA.1 variant, in presence of Brefeldin A that inhibits cytokine secretion into the supernatant and blocks autocrine and paracrine signaling loops. P1-, P2-, and P3-IFN-α production were then assessed. In both conditions, IFN-α was mainly produced by P1-pDC and to a lower extent by P2-pDC (Figure 5D). Additionally, the percentage of IFN-α-producing cells was significantly higher in Delta than Omicron BA.1-stimulated pDC (Figures 5E and S7E). Levels of the proinflammatory cytokines such as IL-6, IL-8, and TNF-α were significantly higher in the supernatant of Omicron BA.1-stimulated than in Alpha B220/95 or Delta-stimulated pDC at the same viral dose (Figures 5F–5H). While Alpha B220/95 and Delta viral variants were only induced substantial levels of TNF-α at the highest SARS-CoV-2 dose (MOI = 4; 50 and 100 pg/mL, respectively), whereas pDC activated with Omicron BA.1 produced 10 to 20-fold higher TNF-α levels (1000 pg/mL at MOI = 4) (Figure 5G). Similar results were observed for IL-6 and IL-8, with Omicron BA.1-stimulated pDC producing significantly higher levels of these cytokines compared to Alpha B220/95 and Delta variants (3- and 12-fold higher levels, respectively, for IL-6 and IL-8). The secretion of these proinflammatory cytokines depended on the magnitude of the viral input.

DC2 stimulated by SARS-CoV-2 variants produced pro-inflammatory cytokines, such as IL-6, IL-8, IL-1β, TNF-α, and IL-12p70 (Figure S8A). IL-6 and IL-1β production was higher in the Omicron BA.1-stimulated DC2 (2- and 7-fold change at the higher assessed viral dose, respectively) as compared to the other viral variants. The secretion tended to be dose-dependent but failed to achieve significance for IL-12p70 and IL-8. We found that Omicron BA.1 induced stronger IL-6 production than Delta, and Delta more strongly than Alpha B220/95, but this result did not extend to other cytokines. TNF-α secretion was dose-dependent. Similar results were obtained for CD88^+^ monocytes exposed to SARS-CoV-2 variants with a dose-dependent production of proinflammatory cytokines IL-1α, IL-6, IL-1β, TNF-α, and smooth response of IL-8 (Figure S8B). We also observed IL-10 secretion only by monocytes stimulated with Omicron BA.1 and Delta at 4 MOI, with no production observed with Alpha B220/95 (Figure S8B). Overall, we established that the P3-pDC subtype generated under Omicron BA.1 stimulation produced more pro-inflammatory cytokines (IL-6, IL-8, and TNF-α) then the P1-pDC subset generated with Alpha B220/95 or Delta SARS-CoV-2.

### СD4^+^ naive T cells underwent a stronger differentiation into robust effector T cells when primed with pDC stimulated with Omicron BA.1

**Figure 6:**
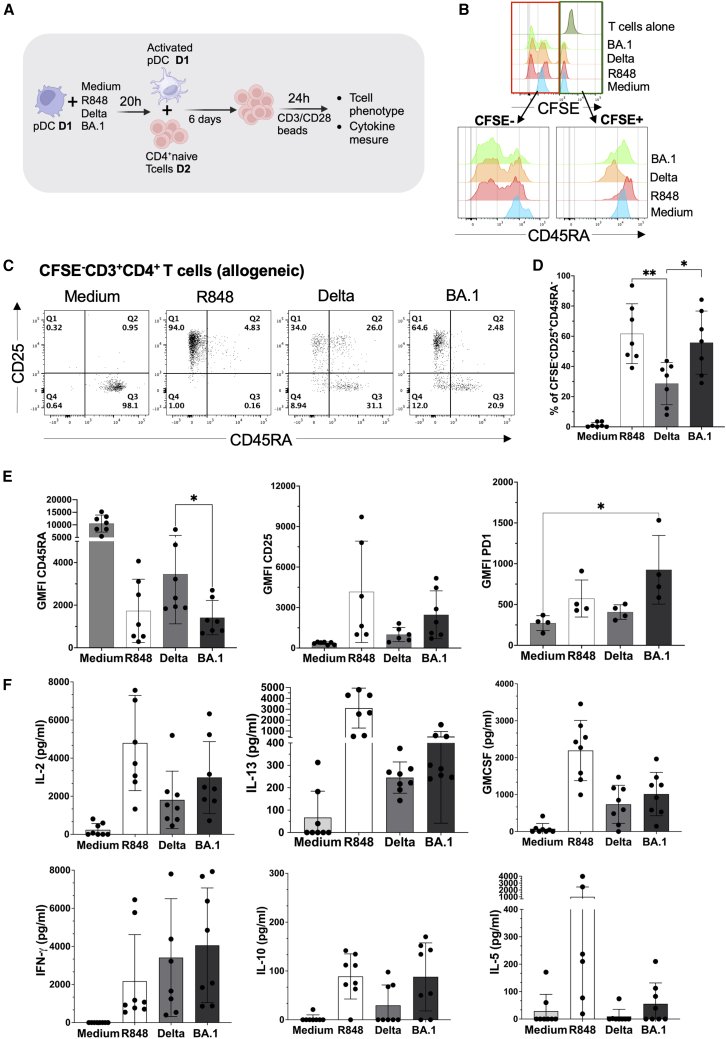
СD4^+^ naive T cells underwent a stronger differentiation into robust effector T cells when primed with pDC stimulated with Omicron BA.1 (A) Experimental schema (Created with BioRender.com). (B) Histogram of CFSE cell trace expression by T cells after 7 days of co-culture, as well as CD45RA expression in CFSE negative and positive population (C). Representative plots of CD3^+^CD4^+^CD25^+^CD45RA^−^ T cells (D). Quantification of CD3^+^CD4^+^CFSE^−^CD25^+^CD45RA^−^ T cells of allogenic co-culture (E). Geometric mean of fluorescence intensity for CD45RA, CD25, PD1 surface markers on T cells (F). IL-2, IL-13, GMCSF, IFN-*γ*, IL-10 and IL-5 cytokine secretion by T cells after 7 days of allogenic co-culture with pDC stimulated with Medium, R848, and Delta or BA.1 SARS-CoV-2 viral variants. *n* = 8 healthy donors from 5 independent experiments. ∗, *p* < 0.05; ∗∗, *p* < 0.01; Mann-Whitney test. Bars represent means ± SD.

Previous studies have shown that besides producing high amounts of IFN, appropriately activated pDC can also function as APCs.^8^ Since we observed phenotypic and functional differences in pDC response to Omicron BA.1 compared to Delta (and Alpha B220/95) SARS-CoV-2 variants, we asked whether there would be any differences in T cell response. CD4^+^ naive T cells (CD45RA^+^CD3^+^CD4^+^CCR7^+^) were isolated from healthy donor (Figure S9A) and cultured with heterologous (in order to increase the proportion of responsive T cells) purified pDC, stimulated with Delta or Omicron BA.1 SARS-CoV-2 variants (Figure 6A). After 7 days of co-culture, we observed a decrease in the CFSE-cell trace signal on the CD4^+^ naive T cell (Figures S9B and S9C) indicating proliferation (Figure 6B). The CFSE-positive T cells retained a naive phenotype with high CD45RA expression. Conversely, the CFSE-negative T cells became CD45RA-negative (Figure 6B) and acquired an effector T cell phenotype (CD3^+^CD4^+^CFSE^−^CD45RA^−^CD25^+^) due to priming by the activated pDC upon challenge with SARS-CoV-2 variants or the positive control (Figure 6C). A 2-fold increase was observed in effector T cells primed with pDC-BA.1 compared to those primed with pDC-Delta (Figure 6D). We observed a significant decrease in CD45RA expression and a significant increase in PD1 expression on T cells primed with pDC-Omicron BA.1 (Figure 6E). T cell secretion of IL-2, IL-13, GM-CSF, IFN-*γ*, IL-10, and IL-5 after 7 days of coculture was measured (Figure 6F). No statistically significant differences in cytokines production were observed between primed pDC-Omicron BA.1 and pDC-Delta T cells. The overall T cell response was Th1-biased. Together, these findings indicate that the SARS-CoV-2 Omicron BA.1 variant primes naive CD4^+^ T cells more efficiently than the Delta variant without qualitative impact on Th cytokines.

## Discussion

The severity of COVID-19 has been reported to be associated with decreased levels of circulating pDC^23^^,^^24^ as well as decreased amount of secreted IFN-α in different compartments,^14^ inborn errors of type I IFN immunity,^25^^,^^26^^,^^27^^,^^28^ or the presence of autoantibodies to IFN-α.^29^ However, these studies have mainly focused on early viral variants—“Wu”, “Delta”, “Alpha”, and “Beta”,—when samples from different patient cohorts were collected and the highest number of severe cases were observed. Nevertheless, the virus has evolved. With the emergence of Omicron, the rate of transmission, the clinical course of the disease and their severity has changed.^30^ Omicron BA.1 is associated with a 61% lower mortality risk and a 56% lower risk of hospitalization.^30^ Nevertheless, the differences in the innate and adaptive immune responses to different viral variants remained unknown.

A key outcome of this study is the discovery that pDC respond differently to different variants *in vitro*, unlike DC2 and monocytes. Here, we report that Omicron BA.1 stimulation favored P3-pDC phenotype characterized by a reduced type I and III IFN response at transcriptional and protein levels, as well as increased production of proinflammatory cytokines. This enabled more efficient naive CD4^+^ T cell priming in an allogeneic co-culture system.

pDC are specialized cells able to produce high levels of type I and III IFN when stimulated by viruses, even without productive infection.^9^^,^^31^^,^^32^ During SARS-CoV-2 infection, they secrete large amounts of IFN-α,^33^^,^^34^ a primary antiviral mucosal molecule that, through ISG activation, hinders the virus from spreading within and outside the organism.^35^^,^^36^ Transcriptomic analysis revealed lower expression of type I and III IFN genes in pDC exposed to the Omicron BA.1 compared to the Delta variant. This is consistent with the observation that Omicron BA.1 preferentially triggers the diversification of pDCs into P3-pDCs subset responsible for T cell priming. In contrast, the Delta and Alpha B220/95 variants predominantly convert pDCs into the P1-pDCs subsets responsible for IFN-α production. It was shown that a low score of IFNI/IFNIII in the nasal mucosa is linked to the amount of the virus in the nose and correlates with the presence of infectious viruses.^37^ Also, the Omicron lineage exhibits increased expression levels of Orf6, a key factor in enhanced innate immune antagonism, making it an important contributor to SARS-CoV-2 transmission.^38^ The lack of innate immune defense, particular type I IFN on the mucosal surfaces, could explain the higher transmissibility of the Omicron BA.1 variant compared to Delta or Alpha B220/95.

One of the main risks of COVID-19 has been the occurrence of a cytokine storm in critically ill patients admitted to intensive care units (ICUs).^39^^,^^40^ It has been suggested that lung-infiltrating macrophages are responsible for producing a cytokine storm associated with high lethality. Hyperactivation of macrophages has been linked to IFN-α production by pDCs upon sensing SARS-CoV-2 via TLR7.^33^ This assumption is also supported by other studies, which found that high levels of IFN-α and IFN- λ2 in the upper and lower respiratory tract were associated with poor clinical outcomes.^14^^,^^15^ Furthermore, despite high type I IFN concentrations in respiratory tissue there was no upregulation of the “protective” ISGs. Therefore, the decrease in disease severity observed with Omicron BA.1 might be associated with the lower IFN-α production by pDC.

The level of TNF-α secreted after pDCs exposure to the SARS-CoV-2 Omicron BA.1 was found to be at least 10-fold higher that observed with the Alpha B220/95 and Delta variants. A recent study suggested that TNF-α can induce the maturation of pDCs through costimulatory molecules and chemokine receptors productions, such as CD80, CD86, HLA-DR, and CCR7.^41^ In addition to TNF-α elevated antigen processing and presentation pathways in mature pDCs, leading to T cell activation.^41^ Therefore, one can speculate that the increased TNF-α level upon stimulation with the Omicron BA.1 variant, could trigger the pDCs to diversify preferentially into the P3-pDC subset. The pDC diversification was shown in response to a single stimulus such as a 24 h exposure to Influenza A/PR/8/34 (H1N1) virus,^8^ leading to P1-, P2-, and P3-pDC subsets with distinct abundances (35%, 50%, and 15%, respectively). The SARS-CoV-2 Alpha B220/95 and 211/61 variants were shown to exhibit a similar trend of pDCs diversification to the Influenza A/PR/8/34 (H1N1) virus with a phenotype of P1-pDC and P2-pDC subsets, the latter being more abundant after exposure to Influenza virus.^9^ The same result was observed with the BetaCoV/France/IDF0571/2020 SARS-CoV-2 variant.^34^ Therefore, stimulation of pDCs by Omicron BA.1 variant favors diversification into the P3-pDCs subset and upregulation of antigen processing and presentation pathways. In contrast, stimulation by the H1N1 virus, Alpha B220/95, BetaCoV, and Delta variants that triggers maturation into IFN-producing pDC subsets.

This differential response of pDC may be either a form of autoregulation whereby the cells themselves adopt an antigen-presenting phenotype in order to destroy the virus, or an intrinsic quality of the SARS-CoV-2 virus and its variants, whereby Omicron BA.1 has evolved to the ability to downregulate IFN production more effectively at the same viral dose as compared to the other variants. The molecular mechanisms involved remain unknown. Viruses are known for their capacity to avoid and subvert type I IFN pathways,^42^ and some SARS-CoV-2 proteins nsp13, nsp14, nsp15, orf6, orf7a, and orf7b were shown to potently inhibit primary IFN production and signaling.^38^^,^^43^^,^^44^

Another possible explanation for the differential response of pDC to SARS-CoV-2 variants is the interaction of the virus with the neuropilin-1 receptor (NRP1). SARS-CoV-2 enters pDC via an ACE2-and TMPRSS2-independent mechanism.^9^^,^^45^ It has been recently demonstrated that the use of anti-NRP1 antibodies impair type I IFN production by pDC.^46^ These findings are consistent with those of another study, predicting that the Spike of the Omicron variant has an increased capacity of binding to NRP1 compared to other variants, favoring higher infectivity of nasal mucosal tissue.^47^ Unlike previous VOCs, SARS-CoV-2 Omicron variants are limited in their ability to generate syncytia upon infection as shown by using a split GFP assay.^45^ This was explained by an alternative SARS-CoV-2 entry via endocytosis involving cathepsins rather than TMPRSS2. It has also been demonstrated that the Spike of Omicron BA.1 promotes a gain in nasal cell infectivity compared to the Delta Spike.^48^

The severity of COVID-19 disease may be explained by the efficiency of the adaptive immune response activation and by the development of a time-variable T cell response to the infection.^49^^,^^50^ To the best of our knowledge, no studies have examined the direct interaction between pDCs and T cells in the cases of SARS-CoV-2 infection, nor the differences in this interaction between variants. In this study we used allogeneic T cells in order to increase the proportion of responder T cells, otherwise using autologous T cells would require being in an Ag-specific system with extremely few naive T cells harboring the required TCR specificity. The more efficient priming of CD4 naive T cells by Omicron BA.1-stimulated pDC compared to Delta-stimulated pDC, shown in this study, may partially account for the above-mentioned difference in the severity of COVID-19 that depends on the SARS-CoV-2 variant.

This study demonstrated for the potential of SARS-CoV-2 VOC to differentially stimulate pDC, leading to distinct patterns of diversification, associated with type I and III IFN production, proinflammatory cytokine production, and the CD4 T cell response. These results may improve our understanding of the significant differences in the severity of COVID-19, in viral transmission, and further illustrate the complexity of the immune response to SARS-CoV-2.

### Limitations of the study

This study has several limitations that should be considered when interpreting the results. All experiments were performed *in vitro* using purified immune cells from healthy donors, which may not fully reflect the complex immune environment during SARS-CoV-2 infection. The lack of clinical samples from COVID-19 patients limits validation of these findings in a physiological context. While the allogeneic T cell co-culture system was used to enhance responder frequency, it does not replicate autologous T cell–pDC interactions. The co-culture experiments may also have been underpowered to detect subtle differences in cytokine responses, especially for certain inflammatory mediators. Moreover, the analysis was restricted to three SARS-CoV-2 variants, and the results may not be generalizable to others. Finally, the 20-h stimulation time point, although suitable for capturing peak pDC responses, may miss earlier or later events in the immune response.

## Resource availability

### Lead contact

Further information and requests for resources and reagents should be directed to and will be fulfilled by the lead contact, Dr Ali Amara (ali.amara@inserm.fr).

### Materials availability

• This study did not generate new unique reagents.
• The reference of the reagents used in this study are available in the key resources table, and upon request to the lead contact.

### Data and code availability

• RNA-seq data have been deposited in the NCBI-based platform “Gene Expression Omnibus” (GEO) database as GSE294888 and are publicly available as of the date of publication, via the following link: https://www.ncbi.nlm.nih.gov/geo/query/acc.cgi?acc=GSE294888.
• This paper does not report original code.
• Any additional information required to reanalyze the data presented in this paper is available from the lead contact upon request.

## Acknowledgments

This work received funding from the ANR-RHU COVIFERON program (ANR-21-RHUS-08) and the HORIZON-HLTH-2021-DISEASE-04 program under grant agreement 101057100 (UNDINE). The authors thank Alessia Zamborlini for critical reading the manuscript.

## Author contributions

A.A. and V.S. designed and coordinated the research. D.K.-E. designed the immunological studies, purified the APC and performed FACS analysis, cytokine production assays, analyzed data, and participated in RNA-seq data analysis. D.T. designed the infection assays, performed the stimulation studies, organized participated in the RNA-seq studies. O.A. performed the RNA-seq bioinformatics data analysis. C.U.-D. performed infection studies on pDC and Vero cells. L.C. produced and titrated virus stocks used in this work. L.M. participated in the functional studies and manuscript preparation. P.T. and J.M. contributed to designer experiments and data analysis. D.K.-E., D.T., A.A., and V.S. wrote the initial manuscript draft and the other authors contributed to its editing in its final form. All authors read and approved the manuscript.

## Declaration of interests

V.S. is a full-time employee at OWKIN, France.

## STAR★Methods

### Key resources table

**Table:** 

REAGENT or RESOURCE	SOURCE	IDENTIFIER
**Antibodies**		

Live/Dead Zombie aqua	Biolegend	Ref. 423102
FITC anti-CD3, monoclonal mouse, clone HIT3a	BD Biosciences	Ref. 555332
FITC anti-CD14, monoclonal mouse, clone TUK4	Miltenyi Biotec	Ref. 130113146
FITC anti-CD16, monoclonal mouse, clone NKP15	BD Bioscience	Ref. 335035
FITC anti-CD19, monoclonal mouse, clone H1B19	Biolegend	Ref. 302205
FITC anti-CD163, monoclonal mouse, clone GHI/61	BD Biosciences	Ref. 563697
FITC anti-CD56, monoclonal mouse, clone HCD56	Biolegend	Ref. 318304
PE-Cy7 anti CD11c, monoclonal mouse, clone Bu15	Biolegend	Ref. 337216
BV650 anti-CD123, monoclonal mouse, clone 6H6	Biolegend	Ref. 306020
APC-vio770 anti-CD2, monoclonal mouse, clone LT2	Miltenyi Biotec	Ref. 130100231
APC anti-CD5, monoclonal mouse, clone L17F12	BD Biosciences	Ref. 555355
PerCp-efluo710 anti-CD1c, monoclonal mouse, cloneL161	Invitrogen	Ref. 46001542
PE anti-CD141, monoclonal mouse, clone AD514H12	Miltenyi Biotec	Ref. 130113318
PE-Dazzle594 anti-CD88, monoclonal mouse, clone S5/1	Biolegend	Ref. 344318
PE anti-CD45RA, monoclonal mouse, clone HI100	BD Biosciences	Ref. 555489
BUV395 anti-CD4 mouse anti-human, clone RPA-T4	BD Biosciences	Ref. 564724
FITC anti-CD3, mouse IgG2aκ, clone BW264/56	Miltenyi Biotec	Ref. 130113128
APC anti-CCR7, recombinant human IgG1, clone REA108	Miltenyi Biotec	Ref. 130120460
BV711 anti-CD123, mouse anti-human	Biolegend	Ref. 306030
BUV737 anti-CD86, monoclonal mouse, clone 2331 (FUN-1)	BD Biosciences	Ref. 612784
PE anti-CD80, monoclonal mouse, clone L307.4	BD Biosciences	Ref. 557227
PE-Cy7 anti-PD-L1, monoclonal mouse, clone 10F.9G2	Biolegend	Ref. 374506
BUV395 anti-HLADR, monoclonal mouse, clone G46-6	BD Biosciences	Ref. 564040
FITC anti-CCR7, monoclonal mouse, clone 150503	BD Biosciences	Ref. 560548
APC anti-CD62L, monoclonal mouse, clone SK11	BD Biosciences	Ref. 4094868
FITC anti-PD-L1, monoclonal mouse, clone MIH1	BD Biosciences	Ref. 558065
PerCP-efluor anti-CD1c, monoclonal mouse, clone L161	Invitrogen	Ref. 46001542
AF700 anti-CD14, monoclonal mouse, clone M5E2	BD Biosciences	Ref. 561029
BV421 anti-CD16, monoclonal mouse, clone 3G8	BD Biosciences	Ref. 562874
BV650 anti-CD80, monoclonal mouse, clone 2D10	Biolegend	Ref. 305227
APC anti-IFN-α, monoclonal mouse, clone LT27:295	Miltenyi Biotec	Ref. 130092602
BUV395 anti-CD4, monoclonal mouse, clone RPA-T4	BD Biosciences	Ref. 564724
BV421 anti-PD1 monoclonal mouse, clone EH12.2H7	Biolegend	Ref. 329919
APC-Cy7 anti-CD25, monoclonal mouse, Clone M-A251	BD Biosciences	Ref. 557753
Mouse monoclonal SARS-CoV/SARS-CoV-2 Nucleocapsid	Sino Biological	Cat #40143-MM05
Alexa Fluor® 647 AffiniPure Donkey Anti-Mouse IgG (H+L)	Jackson ImmunoResearch	Cat #715-605-150

**Bacterial and virus strains**		

SARS-CoV2 Alpha B220/95	Isolated in Saint-Louis Hospital (Paris, France)	ID: EPI_ISL_469284 (220_95)
SARS-CoV2-variant 21A/Delta (B.1.617.2)	Laboratory of Olivier Schwartz	ID: EPI_ISL_2029113
SARS-CoV2-variant Omicron Oμ100.1 p2d4 (Omicron BA.1)	Laboratory of Olivier Schwartz	ID: EPI_ISL _6794907

**Biological samples**		

Cytopheretic rings of healthy donors	Etablissement Francais du sang (French blood bank), Paris, Institut de recherche Saint-Louis	N/A

**Chemicals, peptides, and recombinant proteins**		

Lymphoprep	Proteogenix	Ref. 3824565
Gibco™ RPMI 1640 with GlutaMAX	Thermo Fisher Scientific	Ref. 12027599
MEM nonessential amino acid	Thermo Fisher Scientific	Ref. 11140050
Dulbecco Modified Eagle Medium (DMEM)	Thermo Fisher Scientific	Ref. 41966-029
Hepes buffer solution (1M)	Thermo Fisher Scientific	Ref.15630-056
Glutamax (100X)	Thermo Fischer	Ref. 35050-087
Sodium pyruvate	Thermo Fisher Scientific	Ref. 11360070
Gibco™ Pénicilline-streptomycine (10 000 U/ml)	Thermo Fisher Scientific	Ref. 11548876
TLR7/TLR8 agonist R848 (Resiquimod)	InvivoGen	Ref. tlrl-r848-1
X-VIVO 15 avec Gentamicine Phenol Red avec Transferrine humaine	Lonza	UGAP 4165338
Dynabeads™ Human T-Activator CD3/CD28	Thermo Fisher Scientific	Ref. 11131D
Inhibitor cocktail- 500x, LOT 2290850	Invitrogen	Ref. 00-4980-93
eBeads eBioscience™ UltraComp	Thermo Fisher Scientific	Ref. 01-2222-42
CellTrace™ Yellow Cell Proliferation Kit,	Invitrogen™	Ref. 15584394
32% Paraformaldehyde (formaldehyde) aqueous solution	Electron Microscopy Sciences	Cat #15714
RNAse H	New England BioLabs	Ref. M0297S

**Critical commercial assays**		

Easysep human panDC pre-enrichment kit	StemCell technologies	Ref. 19251
EasySep human Naïve CD4^+^ T cell isolation Kit 2	StemCell technologies	Ref. 17555
Fixation permeabilization buffer eBioscience	Thermo Fisher Scientific	Ref. 88-8824-00
Human IL-12p70 Flex Set	BD Biosciences	Ref. 558283
Human IL-2 Flex Set	BD Biosciences	Ref. 558270
Human IFN-γ Flex Set	BD Biosciences	Ref. 558269
Human IL-10 Flex Set	BD Biosciences	Ref. 558274
Human IL-1β Flex Set	BD Biosciences	Ref. 558279
Human IL-17A Flex Set	BD Biosciences	Ref. 560383
Human TNF Flex Set	BD Biosciences	Ref. 558273
Human IL-6 Flex Set	BD Biosciences	Ref. 558276
Human IL-8 Flex Set	BD Biosciences	Ref. 558277
Human IFN-α Flex Set	BD Biosciences	Ref. 560379
Human IL-13 Flex Set	BD Biosciences	Ref. 558450
IFN-λ1,3 DuoSet ELISA	RD Systems	Ref. DY1598B05
IFN-β DuoSet ELISA	RD Systems	Ref. DY81405
Maxima First Strand cDNA Synthesis Kit	Thermo Fisher Scientific	Cat #K1671
RNeasy mini kit	Qiagen	Cat #74106
Power SYBRgreen PCR Master mix	Applied Biosystems	Ref. 4367659

**Deposited data**		

RNA-seq raw and analysed data	This paper	GSE294888 https://www.ncbi.nlm.nih.gov/geo/query/acc.cgi?acc=GSE294888

**Experimental models: Cell lines**		

Vero E6	ATCC	Cat #CRL-1586

**Oligonucleotides**		

E_Sarbeco Forward primer ACA GGT ACG TTA ATA GTT AAT AGC GT	Corman et al.^51^	N/A
E_Sarbeco Reverse primer ATA TTG CAG CAG TAC GCA CAC A	Corman et al.^51^	N/A
QuantiTect RT-PCR Primer assay HS_GAPDH	Qiagen	QT01192646

**Software and algorithms**		

FlowJo software (version 10.10)	BD Biosciences	https://flowjo.com/flowjo10/download
FCAP array software	BD Biosciences	https://www.bdbiosciences.com/en-at/products/instruments/software-informatics/instrument-software/fcap-array-software-v3-0.652099?tab=product_details
Prism 9.5	GraphPad	https://www.graphpad.com/updates/prism-910-release-notes
Attune NxT software	Thermo Fisher Scientific	Version 2.6
R software	The Comprehensive R archive network	Version 4.3.2
pheatmap	Raivo Kolde	https://cran.r-project.org/web/packages/pheatmap/index.html
EnhancedVolcano	Kevin Blighe	https://github.com/kevinblighe/EnhancedVolcano
ComplexHeatmap	Zuguang Gu	https://www.bioconductor.org/packages/release/bioc/html/ComplexHeatmap.html
ClusterProfiler	Guangchuang Yu, Li-Gen Wang	https://www.bioconductor.org/packages//2.13/bioc/html/clusterProfiler.html
Kallisto	Pachter laboratory	https://pachterlab.github.io/kallisto/
Tximport	Michael Love	https://www.bioconductor.org/packages/release/bioc/html/tximport.html
DESeq2	Michael Love	https://www.bioconductor.org/packages//2.13/bioc/html/DESeq2.html
GSVA	Robert Castelo	https://www.bioconductor.org/packages/release/bioc/html/GSVA.html

**Other**		

LightCycler 480	Roche	N/A
Attune NxT	Thermo Fisher Scientific	N/A

### Experimental model and study participant details

#### Cell lines

Vero E6 cells (African green monkey kidney cells) were maintained in Dulbecco Modified Eagle Medium (DMEM; Invitrogen Life Technologies) supplemented with 10% fetal bovine serum, 1% GlutaMAX and 1% penicillin/streptomycin (P/S) (Life Technologies). This cell line was regularly tested for the absence of mycoplasma contamination. pDC, DC2 and monocytes used in this study, and isolated from cytopheretic rings of healthy donors, were characterized in the method details section.

### Method details

#### Cell isolation and culture

Cytopheretic rings of healthy donors were obtained from Etablissement Francais du sang (French blood bank, Institut de recherche Saint-Louis, Paris, France). The number of healthy donors was reported in the figure’s legend (when applicable) and isolated cells from each donor were subjected to the same stimulation conditions. The PBMCs were isolated through Ficoll density gradient centrifugation (Ficoll-Paque, GE Healthcare) at 2000 rpm for 20 min without braking. Easysep human panDC pre-enrichment kit (StemCell technologies, ref. 19251) was used for primary DC magnetic sorting cell isolation, followed by flow cytometry cell sorting on BD FACSARIA 3 cell sorter. pDC and DC2 were sorted on the basis of Live, lineage^-^ (CD3, CD14, CD16, CD19, CD20, CD56, CD163) and specific dendritic cells markers (CD11c^-^, CD123^+^, CD2^-^, CD5^-^ for pDC, and CD11c^+^, CD1c^+^, CD141^-^ for DC2). Human monocytes were directly isolated from PBMC through flow cytometry cell sorting on BD FACS ARIA 3 cell sorter on the basis of Live, Lineage^-^ (CD3, CD19, CD20, CD56, CD163), and specific monocyte marker CD88^+^. The purity of isolated immune cell populations was >98% (Figure S3). The purity was assessed by passing sorting cell on flow cytometry right there, after isolation (Gating strategy and purity in Figures S3 and S5).

Cells were stained with Live/Dead Zombie aqua (BV510, Ref. 423102, Biolegend), FITC anti-CD3 (clone HIT3a, Ref. 555332, BD Biosciences), FITC anti-CD14 (clone TUK4, Ref. 130113146, Miltenyi Biotec), FITC anti-CD16 (clone NKP15, Ref. 335035, BD Bioscience), FITC anti-CD19 (clone H1B19, Ref. 302205, Biolegend), FITC anti-CD163 (clone GHI/61, Ref. 563697, BD Biosciences), FITC anti-CD56 (clone HCD56, Ref. 318304, Biolegend ), PE-Cy7 anti-CD11c (clone Bu15, Ref. 337216, Biolegend), BV650 anti-CD123 (clone 6H6, Ref. 306020, Biolegend), APC-vio770 anti-CD2 (Ref. 130100231, Miltenyi Biotec), APC anti-CD5 (Ref. 555355, BD Biosciences), PerCp-efluo710 anti-CD1c (cloneL161, Ref. 46001542, Invitrogen), PE anti-CD141 (clone AD514H12, Ref. 130113318, Miltenyi Biotec), PE-Dazzle594 anti-CD88 (clone S5/1, Ref. 344318, Biolegend).

Human naïve CD4^+^ T cells were isolated from PBMCs of healthy donors through EasySep human Naïve CD4^+^ T cell isolation Kit 2 (ref 17555) with the purity >90%. The purity was assessed by passing sorting cell on BD LSR Fortessa™ Flow Cytometer (BD Biosciences) after isolation, based on the following markers: Live/Dead Zombie aqua (BV510, Biolegend, Ref. 423102), PE anti-CD45RA (BD Biosciences, Ref. 555489), BUV395 anti-CD4 (clone RPA-T4, BD Biosciences, Ref. 564724), FITC anti-CD3 (clone BW264/56, Miltenyi Biotec, Ref. 130113128), APC anti-CCR7 (clone REA108, Miltenyi Biotec, Ref. 130120460).

pDC, DC2 and monocytes were cultured in medium RPMI 1640 with GlutaMAX, 10% of FBS, 1% of MEM nonessential amino acid, 1% of sodium pyruvate and 1% of penicillin-streptomycin.^9^

Since no information regarding the donors were provided by the “Etablissement Francais du sang” (French blood bank, Institut de recherche Saint-Louis, Paris, France), the sex of the isolated pDC, DC2 and monocytes used in this study was unknown.

#### Viruses

SARS-CoV-2 viral variant Alpha B220/95, 21A/Delta (B.1.617.2) and Omicron BA.1 were isolated as reported previously.^9^ SARS-CoV2 Delta and Omicron BA.1 variants were obtained from Pr Olivier Schwartz lab (Pasteur Institute) and have been previously characterized.^52^ SARS-CoV-2 strains were amplified on Vero E6 in DMEM-2% (DMEM supplemented with 2% FBS, 1% penicillin-streptomycin, 1% GlutaMAX, and 25 mM Hepes). Viruses were passaged three times before being used for experiments. Viruses were purified through a 20% sucrose cushion by ultracentrifugation at 80,000 g for 2h at 4°C and pellets were resuspended in HNE 1X, pH 7.4 (Hepes 5 mM, NaCl 150 mM, and EDTA 0.1 mM), aliquoted, and stored at −80°. Virus titers were determined by plaque assays in Vero E6 cells and expressed as Plaque Forming Unit per milliliter (PFU/ml). To titer the viruses, 10-fold dilution of viral stocks were added to the cells and incubation for 1h at 37°C, in a 5% CO2 atmosphere, was performed to allow virus adsorption. The inoculum was replaced with Avicel 2.4% mixed at an equal volume with DMEM supplemented with 4% FBS, 2% Glutamax, 50 mM MgCl2, and 0.225% of NaHCO3, and incubated 3 d at 37°C before plaque counting. Virus titers were also performed using a direct RT-qPCR method as described.^53^

#### Stimulation assay of pDC, DC2 and monocytes with SARS-CoV-2 viral variants

In order to stimulate freshly sorted pDCs, DC2s and monocytes, 1 x 10^5^ cells were plated in V-bottom 96-well plates in presence of medium alone (RPMI 1640 medium with GlutaMAX, 10% FBS, 1% of MEM nonessential amino acid, 1% of sodium pyruvate, and 1% of penicillin-streptomycin), the TLR7/TLR8 agonist R848 (Resiquimod), SARS-CoV-2 strain 220/95, Delta or Omicron BA.1, at MOI=0.4 and 4. After 20 h of culture at 37°C, in a 5% CO2 atmosphere, cells supernatants were collected and stored at -80°C for cytokine level assessment while stimulated cells were stained for flow cytometry analysis.

#### Infection assays

Vero cells were plated at a density of 50,000 cell per well in 24-well plates for 4 h before adding SARS-CoV-2 inoculum, diluted in DMEM-2%. Freshly purified pDCs were seeded in 96-well plates at a density of 50,000 cells per well and incubated with SARS-CoV-2 diluted in RPMI 1640 medium supplemented with GlutaMAX, 10% of FBS, 1% of MEM nonessential amino acid, 1% of sodium pyruvate, and 1% of penicillin-streptomycin. At 48 h after inoculation, Vero cells were detached and transferred to 96-well plates. Vero and pDCs were washed with PBS and fixed with 2% (vol/vol) paraformaldehyde diluted in PBS for 10 min at room temperature. Cells were incubated for 1h at 4°C with a monoclonal antibody, targeting SARS-CoV-2 nucleoprotein, at a concentration of 1 μg/ml (40143-MM05; Sino Biological) diluted in permeabilization flow cytometry buffer (PBS supplemented with 5% FBS, 0.5% [wt/vol] saponin, and 0.1% sodium azide). After washing with flow cytometry buffer, cells were incubated with 1 μg/ml of Alexa Fluor 647-conjugated (715-605-150; Jackson ImmunoResearch) Donkey anti-mouse IgG diluted in permeabilization flow cytometry buffer for 30 min at 4°C. SARS-CoV-2 infection was quantified by flow cytometry using an Attune NxT Flow Cytometer (Thermo Fisher Scientific) for the acquisition of the data and FlowJo software for the analysis.

In parallel, quantitative RT-PCR was used to assess the presence of viral genome at 48h post infection. In P96 plate, 30,000 cells (Vero or pDC) were seeded an infected at a MOI of 1 and 5 using SARS-CoV-2 variants Delta and Omicron BA.1. After 48h post infection, total RNA from each condition was extracted using the RNeasy plus mini kit (Qiagen) according to manufacturer’s instruction. Furthermore, cDNAs were obtained from 400 ng total RNA by using the Maxima First Strand Synthesis Kit following manufacturer’s instruction (Thermo Fisher Scientific). Amplification products were treated with 1 Unit of RNAse H for 20 min at 37°C, followed by 10 min at 72°C for enzyme inactivation. cDNAs were diluted in one third in RNAse free water before quantification. The E_Sarbeco primers described in Corman et al., 2020^51^ (Forward primer: ACA GGT ACG TTA ATA GTT AAT AGC GT and Reverse primer ATA TTG CAG CAG TAC GCA CAC A) were used for viral RNA quantification and Quantitect primers for GAPDH RNA quantification were acquired form Qiagen. Quantitative PCR was performed on a LightCycler 480 (Roche).

#### RNA-seq preparation and sequencing

RNA-seq analysis on the pDC after 20h of infection with SARS-CoV-2 viral variants was performed by Paris Brain Institute, ICM, Hôpital de la Pitié-Salpêtrière, Plateforme de Genotypage/Séquençage. The samples were checked for activation before RNA-seq analysis, with extracellular staining of activation markers. ARN extraction was performed using RNeasy mini kit (Qiagen) according to the manufacturer’s protocol.

#### RNA-seq analysis

All samples were assessed for quality using the FastQC (Babraham Bioinformatics, 2010) package with MutliQC. All downstream analyses were performed in R 4.3.2, using Rstudio. The abundance of transcripts was fetched with the kallisto program from Pachter. Our data was mapped to the genome using the tximport package. The differential gene expression analyses were conducted with the DESeq2 package. Genes with an FDR below 0.05 were retained. The absolute log2 fold change cutoff was set at 1, meaning that only genes with a fold change above 2 or below -2 were kept. The gene functional annotation was performed using the GSVA package (for Figures 1F and 2B) and the ClusterProfiler package using differentially expressed genes retrieved from DESeq2 (for Figure 2C). We generated heatmaps with the ComplexHeatmap and pheatmap packages and the volcano plots were obtained with the Enhanced Volcano package.

#### Flow cytometry analysis

After stimulation the pDC, DC2 and monocytes were analyzed by flow cytometry on BD LSR Fortessa™ Flow Cytometer (BD Biosciences). Cells were stained with Live/Dead Zombie aqua (BV510, Biolegend, Ref. 423102), BV711 anti-CD123 (Biolegend, ref. 306030), BUV737 anti-CD86 (BD Biosciences, ref. 612784), PE anti-CD80 (BD Biosciences, ref. 557227), PE-Cy7 anti-PD-L1 (Biolegend, ref. 374506), BUV395 anti-HLADR (BD Biosciences, ref. 564040), FITC anti-CCR7 (BD Biosciences, ref. 560548), APC anti-CD62L (BD Biosciences, Ref. 4094868), FITC anti-PD-L1 (BD Biosciences, clone MIH1, Ref. 558065), PerCP-efluor anti-CD1c (Invitrogen, ref. 46001542), APC anti-CCR7 (Miltenyi biotec, clone REA108, Ref. 130120460), AF700 anti-CD14 (BD Biosciences, clone M5E2, Ref. 561029), BV421 anti-CD16 (BD Biosciences, clone 3G8, Ref. 562874), BV650 anti-CD80 (Biolegend, clone 2D10, Ref. 305227).

For intracellular staining, after 16 h of stimulation the pDC were treated with brefeldin A for 5 hours. Cells were stained with Live/Dead, surface staining and then treated with fixation permeabilization buffer eBioscience (ThermoFicher, Ref. 88-8824-00) according to the manufacturer’s protocol, followed by intracellular staining with APC anti-IFN-α (Miltenyi Biotec, Ref. 130092602).

Subsequent data analysis was performed on FlowJo software (version 10.10). Flow cytometry analyses were performed at the flow cytometry core facility of Institut de Recherche Saint-Louis (Paris, France).

#### pDC–naive CD4^+^T cell co cultures

CD4^+^ naïve T cells, stained with CFSE cell trace, were cultured for 6 days with allogeneic activated pDC (R848 or Delta/Omicron BA.1 SARS-CoV-2 viral variants) at a ratio of 5:1 in X-Vivo medium. After coculture, T cell expansion was determined by cell counting and Flow cytometry analysis. Supernatants were collected after 24h of polyclonal restimulation with anti-CD3/CD28 microbeads (Dynal). T cell numbers were normalized to 10^6^ cells/ml for restimulation. Cytokine measurements were performed with a BD cytometric bead array according to the manufacture’s protocol. T cell proliferation and activation were assessed with CFSE cell trace and extracellular activation markers using LSR Fortessa (BD Biosciences). T cells were stained with Live/Dead Zombie aqua (BV510, Biolegend, Ref. 423102), PE-anti-CD45RA (BD Biosciences, Ref. 555489), BUV395 anti-CD4 (BD Biosciences, clone RPA-T4, Ref. 564724), BV421 anti-PD1 (Biolegend, Ref. 329919), APC-Cy7 anti-CD25 (BD Biosciences, Ref. 557753).

#### Cytokines analysis

pDC, DC2, monocytes and T cell cytokine production of IFN-α2, IFN-γ, IL-6, IL-8, IL-13, IL-2, IL-1β, TNF-α, IL-10, IL-12p70, IL-5, IL-4, GM-CSF, IL-17A, was measured in culture supernatants using BD cytometric bead array according to the manufacture’s protocol, with a 20pg/ml detection limit. Acquisition was performed on a LSR Fortessa (BD Biosciences), and cytokine concentrations were determined with FCAP array software ((BD Biosciences). The concentration of secreted IFN-λ1,3 and IFN-β were measured by ELISA (DuoSet, RD Systems, Ref DY1598B05 and DY81405 respectively) according to manufacture protocol. The optical density and concentrations were determined with spectrophotometer.

### Quantification and statistical analysis

Statistical analyses were performed using Prism9.1 software (GraphPad Software). Non-parametric two-tailed Mann-Whitney test was used. Two-way Anova test with Geisser-Greenhouse correction was used where indicated. p-values below 0.05 were considered to indicate statistically significant differences.
