# Supplementary material for: Differential response of human plasmacytoid pre-dendritic cells to SARS-CoV-2 variants
Source: iScience. 2025 Aug 18;28(9):113394. doi: 10.1016/j.isci.2025.113394 (PMC12441710; doi:10.1016/j.isci.2025.113394)
Supplement: Document S1. Figures S1–S9 [file mmc1.pdf]

iScience, Volume *28*

Supplemental information

Differential response of human plasmacytoid pre-dendritic cells to SARS-CoV-2 variants

Daria Kartasheva-Ebertz, Dimitrios Topalis, Claudia Umana-Diaz, Okan Ayas, Laurine Couture, Pierre Tonnerre, Jasna Medvedovic, Laurent Meertens, Vassili Soumelis, and Ali Amara

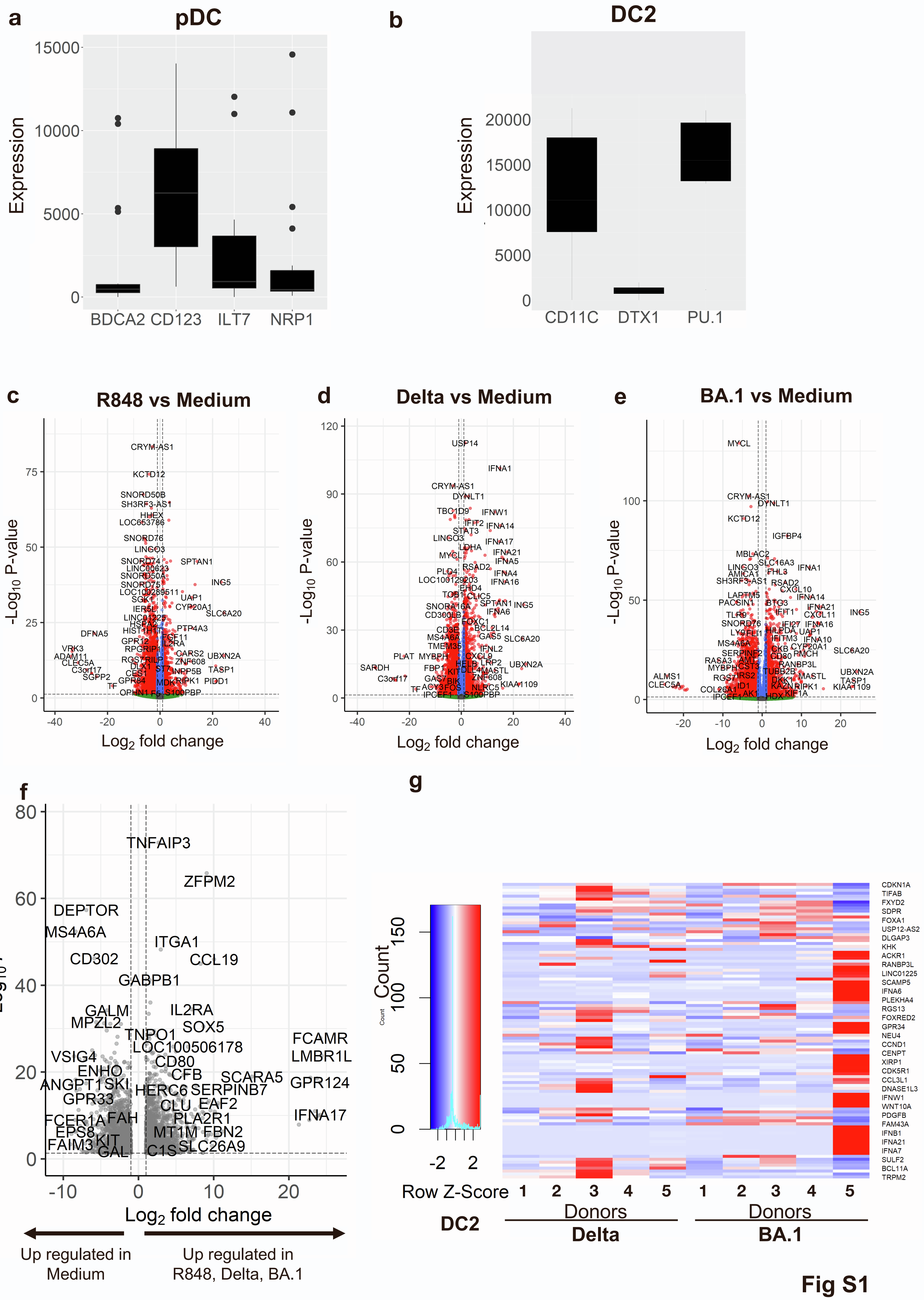
a
pDC
Expression
15000
10000
5000
0
BDCA2 CD123 ILT7 NRP1
b
DC2
Expression
20000
15000
10000
5000
0
CD11C DTX1 PU.1
c
R848 vs Medium
d
Delta vs Medium
e
BA.1 vs Medium
$-Log_{10}$ P-value
Log_2 fold change
f
$-Log_{10}$ P
Log_2 fold change
TNFAIP3
ZFPM2
DEPTOR
MS4A6A
ITGA1
CD302
CCL19
GABPB1
GALM
IL2RA
MPZL2
SOX5
TNPO1
LOC100506178
FCAMR
LMBR1L
VSIG4
CD80
ENHO
CFB
SCARA5
ANGPT1
SKI
GPR124
HERC6
SERPINB7
GPR33
CLU
EAF2
FCER1A
FAH
PLA2R1
IFNA17
EPS8
MT1M
FBN2
KIT
FAIM3
C1S
SLC26A9
GAL
Up regulated in Medium
Up regulated in R848, Delta, BA.1
g
Count
Row Z-Score
DC2
1 2 3 4 5
Donors
Delta
1 2 3 4 5
Donors
BA.1
CDKN1A
TIFAB
FXYD2
SDPR
FOXA1
USP12-AS2
DLGAP3
KHK
ACKR1
RANBP3L
LINC01225
SCAMP5
IFNA6
PLEKHA4
RGS13
FOXRED2
GPR34
NEU4
CCND1
CENPT
XIRP1
CDK5R1
CCL3L1
DNASE1L3
IFNW1
WNT10A
PDGFB
FAM43A
IFNB1
IFNA21
IFNA7
SULF2
BCL11A
TRPM2

Fig S1

Figure S1 RNAseq analysis of pDC and DC2 purified cell populations, stimulated for 20h by Medium, R848, Delta or BA.1 SARS-CoV-2 viral variants. **a**. Quality control gene expression for pDC cells. **b**.Quality control gene expression for DC2 cells. **c**. Volcano plot of differentially expressed genes in pDC stimulated with R848 vs Medium. **d**. Volcano plot of differentially expressed genes in pDC stimulated with Delta vs Medium. **e**. Volcano plot of differentially expressed genes in pDC stimulated with BA.1 vs Medium. **f**. Volcano plot of differentially expressed genes in DC2 stimulated with R848, Delta or BA.1 SARS-CoV-2 variants vs non stimulated (Medium). **g**. HeatMap of 100 up regulated DEGs in DC2 stimulated by Delta vs BA.1.

a

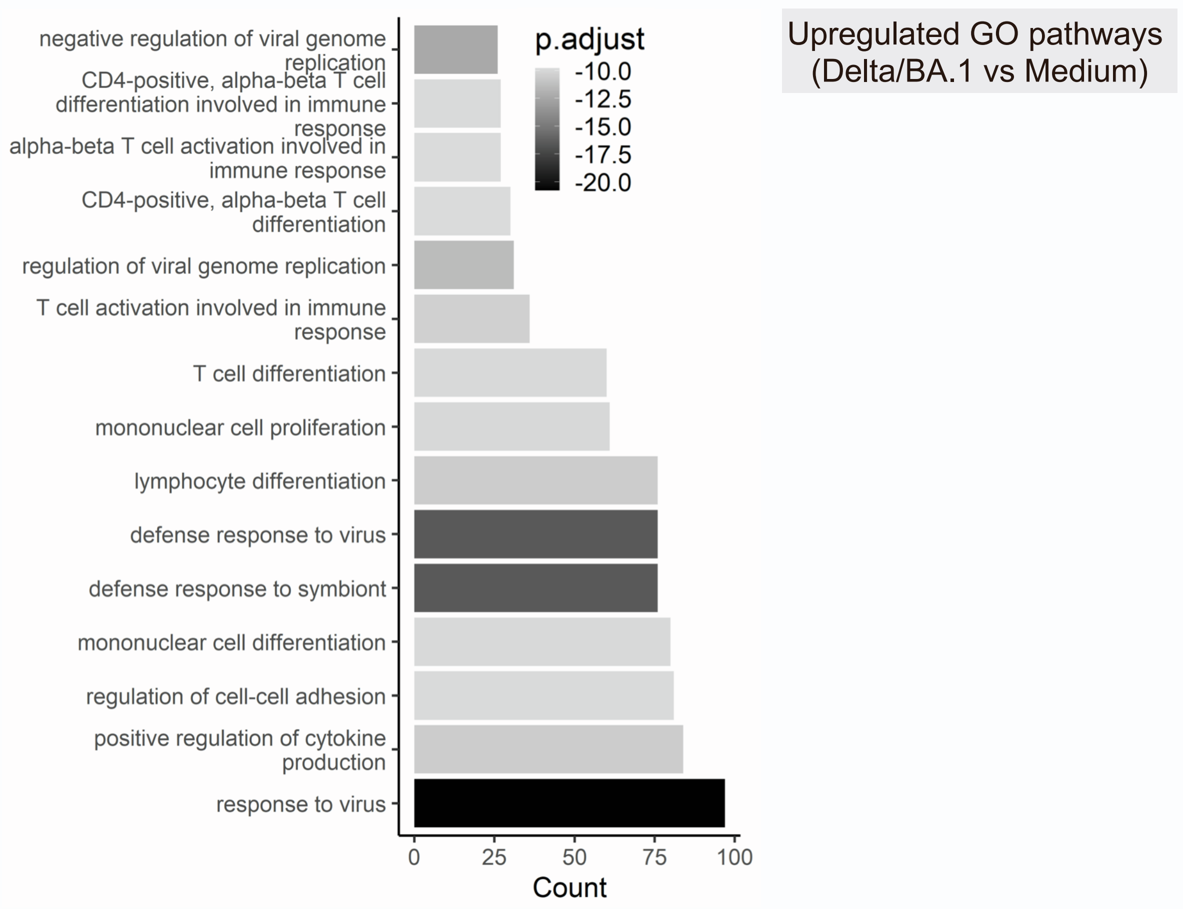
Upregulated GO pathways
(Delta/BA.1 vs Medium)
p.adjust
-10.0
-12.5
-15.0
-17.5
-20.0
negative regulation of viral genome replication
CD4-positive, alpha-beta T cell differentiation involved in immune response
alpha-beta T cell activation involved in immune response
CD4-positive, alpha-beta T cell differentiation
regulation of viral genome replication
T cell activation involved in immune response
T cell differentiation
mononuclear cell proliferation
lymphocyte differentiation
defense response to virus
defense response to symbiont
mononuclear cell differentiation
regulation of cell-cell adhesion
positive regulation of cytokine production
response to virus
0
25
50
75
100
Count

b

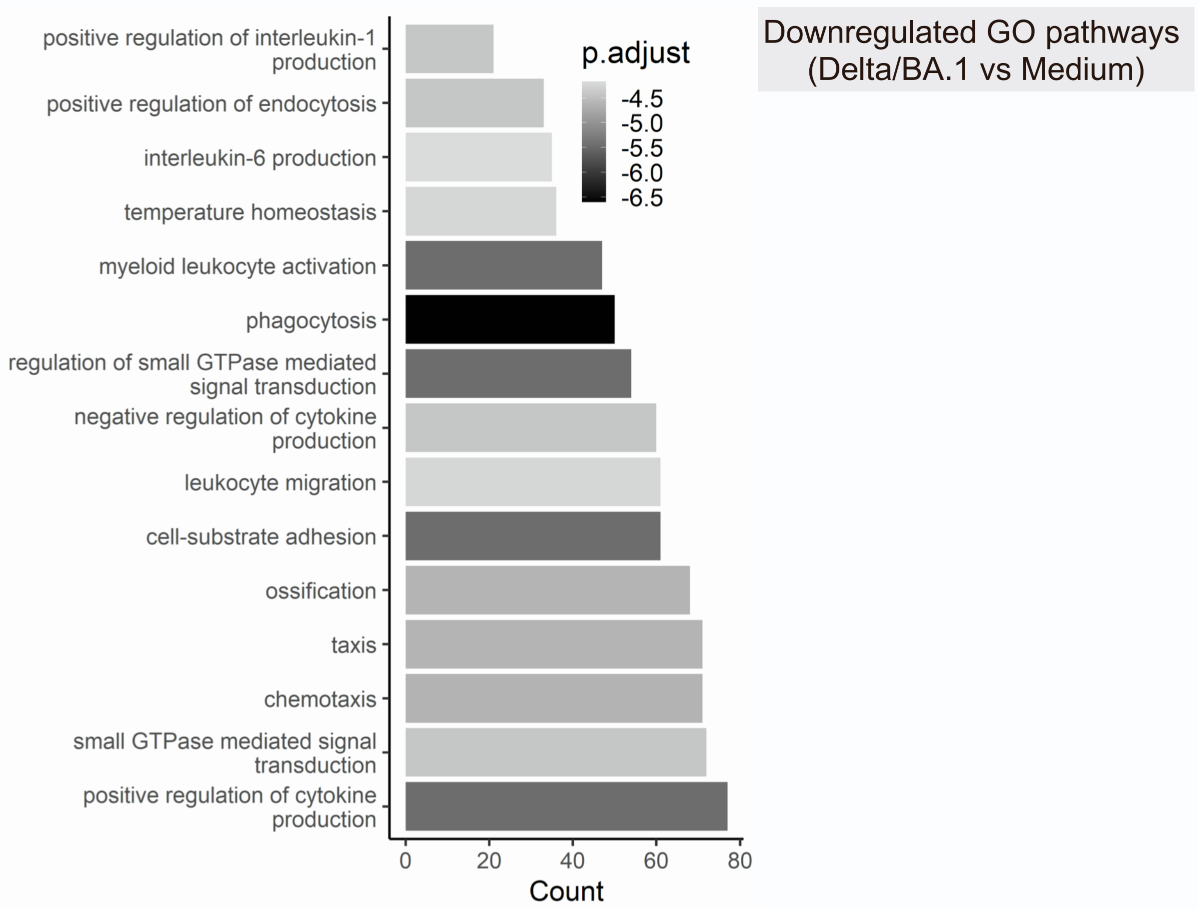
Downregulated GO pathways
(Delta/BA.1 vs Medium)
p.adjust
-4.5
-5.0
-5.5
-6.0
-6.5
positive regulation of interleukin-1 production
positive regulation of endocytosis
interleukin-6 production
temperature homeostasis
myeloid leukocyte activation
phagocytosis
regulation of small GTPase mediated signal transduction
negative regulation of cytokine production
leukocyte migration
cell-substrate adhesion
ossification
taxis
chemotaxis
small GTPase mediated signal transduction
positive regulation of cytokine production
0
20
40
60
80
Count

Fig S2

Figure S2 Up and Down regulated GO pathways in DC2 cells.

a. Up regulated GO pathways Stimulated vs Non stimulated **b**. Down regulated Go pathways Stimulated vs Non stimulated RNAseq analysis.

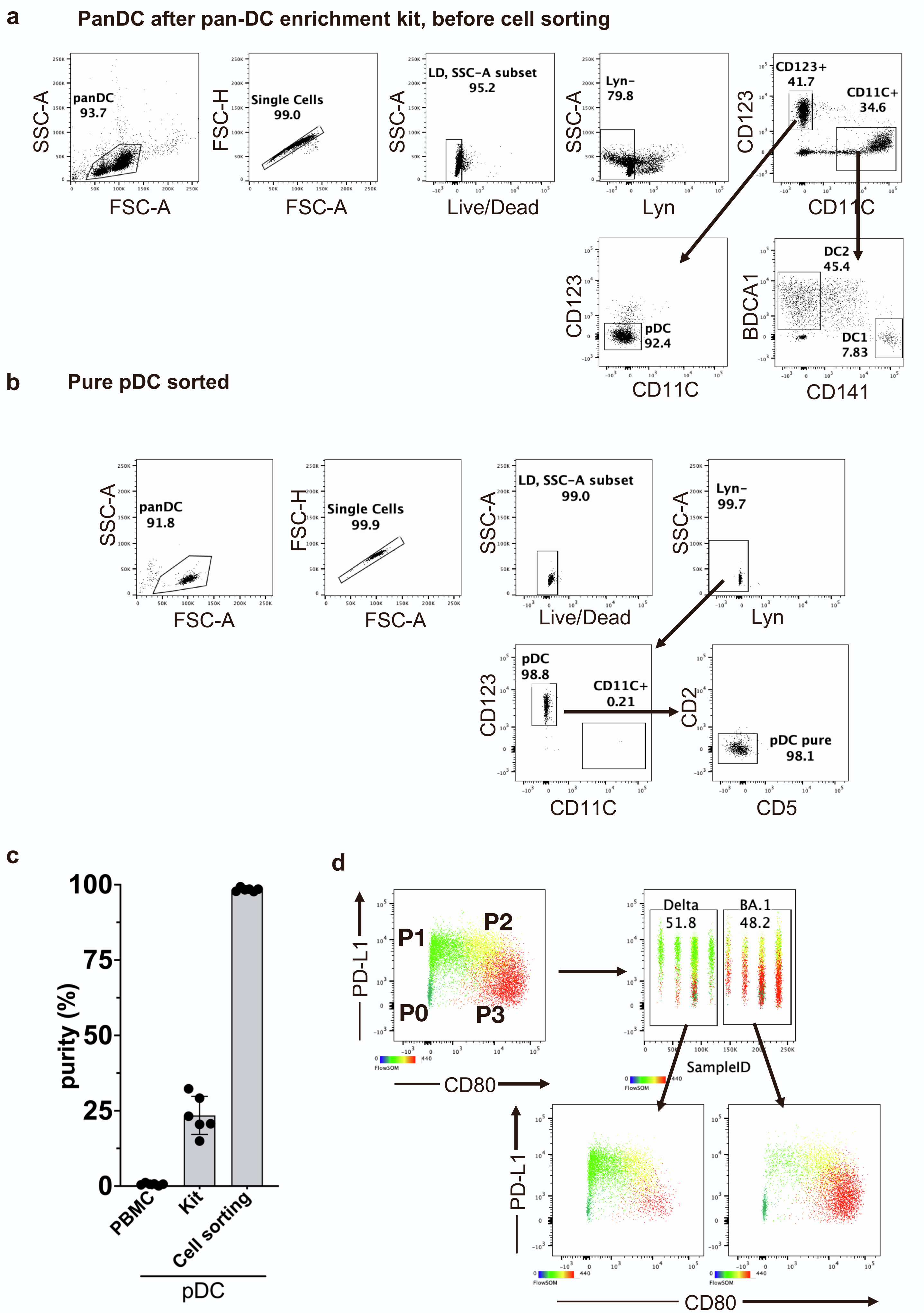

a
PanDC after pan-DC enrichment kit, before cell sorting
panDC 93.7
Single Cells 99.0
LD, SSC-A subset 95.2
Lyn- 79.8
CD123+ 41.7
CD11C+ 34.6
pDC 92.4
DC2 45.4
DC1 7.83
SSC-A
FSC-A
FSC-H
Live/Dead
Lyn
CD123
CD11C
BDCA1
CD141
b
Pure pDC sorted
panDC 91.8
Single Cells 99.9
LD, SSC-A subset 99.0
Lyn- 99.7
pDC 98.8
CD11C+ 0.21
pDC pure 98.1
CD2
CD5
c
purity (%)
100
75
50
25
0
PBMC
Kit
Cell sorting
pDC
d
P1
P2
P0
P3
Delta 51.8
BA.1 48.2
PD-L1
CD80
SampleID
FlowSOM

Fig S3.

Figure S3 Purity and Gating strategy of pDC and DC2 isolation.

a. Gating strategy for pDC and DC2 cell sorting (ARIA3) from panDC population after pan-DC enrichment kit. Representative plots of Flow Cytometry analysis **b**. Purity of sorted pDC. c. DotPlot generated by unsupervised analysis FlowSom on 8 concatenated samples of purified pDCs from the blood of healthy donors, stimulated by Delta and BA.1 SARS-CoV-2 viral variants for 20 hours .

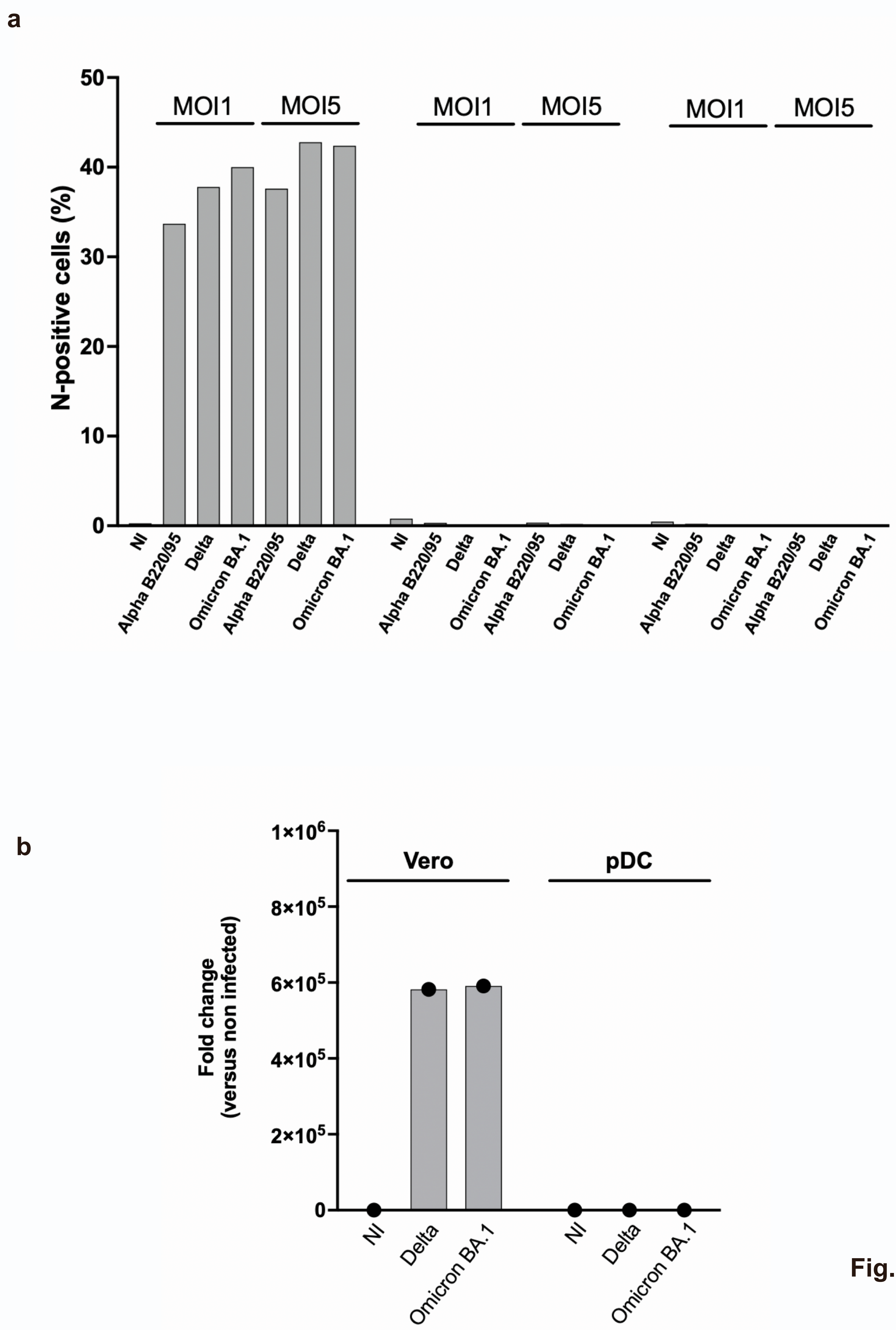
a
N-positive cells (%)
50
40
30
20
10
0
MOI1
MOI5
MOI1
MOI5
MOI1
MOI5
NI
Alpha B220/95
Delta
Omicron BA.1
Alpha B220/95
Delta
Omicron BA.1
NI
Alpha B220/95
Delta
Omicron BA.1
Alpha B220/95
Delta
Omicron BA.1
NI
Alpha B220/95
Delta
Omicron BA.1
Alpha B220/95
Delta
Omicron BA.1
b
Fold change (versus non infected)
1×10^6
8×10^5
6×10^5
4×10^5
2×10^5
0
Vero
pDC
NI
Delta
Omicron BA.1
NI
Delta
Omicron BA.1

Fig. S4

Figure S4 Viral infection of pDC and DC2

a. Column chart representation of the percentage of vero cell, pDC and DC2 positive for SARS-CoV-2 N protein. b. Quantification of viral RNA by RT-PCR after infection of vero cells and pDC using SARS-CoV2 variant Delta and Omicron BA.1 at MOI=5, These graphs were generated using Prism Graphpad version 9.5.

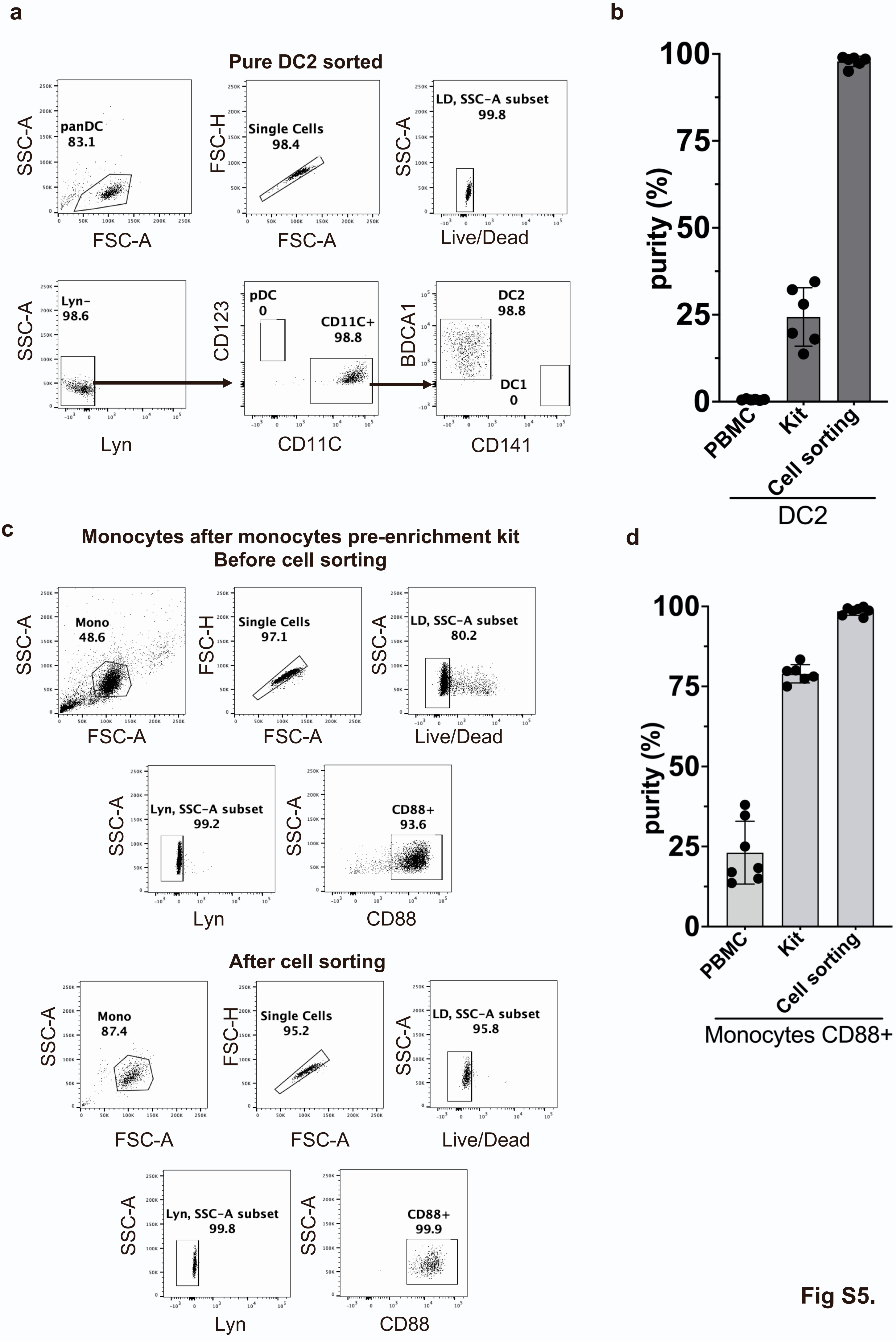
a
Pure DC2 sorted
panDC
83.1
SSC-A
FSC-A
Single Cells
98.4
FSC-H
FSC-A
LD, SSC-A subset
99.8
SSC-A
Live/Dead
Lyn-
98.6
SSC-A
Lyn
pDC
0
CD11C+
98.8
CD123
CD11C
DC2
98.8
DC1
0
BDCA1
CD141
b
purity (%)
100
75
50
25
0
PBMC
Kit
Cell sorting
DC2
c
Monocytes after monocytes pre-enrichment kit
Before cell sorting
Mono
48.6
SSC-A
FSC-A
Single Cells
97.1
FSC-H
FSC-A
LD, SSC-A subset
80.2
SSC-A
Live/Dead
Lyn, SSC-A subset
99.2
SSC-A
Lyn
CD88+
93.6
SSC-A
CD88
After cell sorting
Mono
87.4
SSC-A
FSC-A
Single Cells
95.2
FSC-H
FSC-A
LD, SSC-A subset
95.8
SSC-A
Live/Dead
Lyn, SSC-A subset
99.8
SSC-A
Lyn
CD88+
99.9
SSC-A
CD88
d
purity (%)
100
75
50
25
0
PBMC
Kit
Cell sorting
Monocytes CD88+

Fig S5.

Figure S5 DC2 purity and Gating strategy of monocyte isolation.
a. Purity of sorted DC2. b. DC2 purity quantification c. Gating strategy for monocytes cell sorting based on CD88+ cells after monocytes pre-enrichment kit. d. Purity quantification of CD88^{+} monocytes.

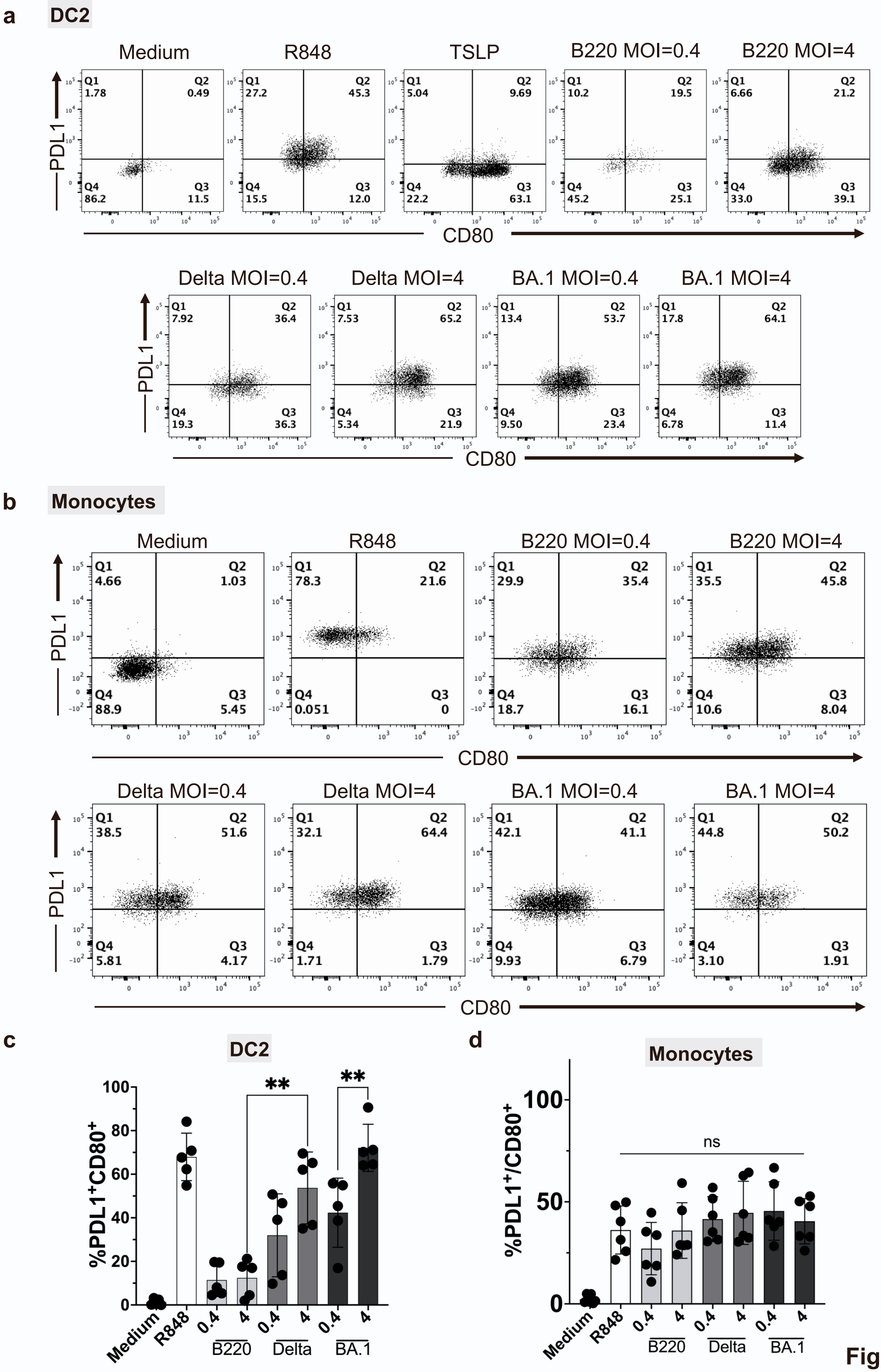
a
DC2
Medium
Q1 1.78 Q2 0.49 Q4 86.2 Q3 11.5
R848
Q1 27.2 Q2 45.3 Q4 15.5 Q3 12.0
TSLP
Q1 5.04 Q2 9.69 Q4 22.2 Q3 63.1
B220 MOI=0.4
Q1 10.2 Q2 19.5 Q4 45.2 Q3 25.1
B220 MOI=4
Q1 6.66 Q2 21.2 Q4 33.0 Q3 39.1
PDL1
CD80
Delta MOI=0.4
Q1 7.92 Q2 36.4 Q4 19.3 Q3 36.3
Delta MOI=4
Q1 7.53 Q2 65.2 Q4 5.34 Q3 21.9
BA.1 MOI=0.4
Q1 13.4 Q2 53.7 Q4 9.50 Q3 23.4
BA.1 MOI=4
Q1 17.8 Q2 64.1 Q4 6.78 Q3 11.4
PDL1
CD80
b
Monocytes
Medium
Q1 4.66 Q2 1.03 Q4 88.9 Q3 5.45
R848
Q1 78.3 Q2 21.6 Q4 0.051 Q3 0
B220 MOI=0.4
Q1 29.9 Q2 35.4 Q4 18.7 Q3 16.1
B220 MOI=4
Q1 35.5 Q2 45.8 Q4 10.6 Q3 8.04
PDL1
CD80
Delta MOI=0.4
Q1 38.5 Q2 51.6 Q4 5.81 Q3 4.17
Delta MOI=4
Q1 32.1 Q2 64.4 Q4 1.71 Q3 1.79
BA.1 MOI=0.4
Q1 42.1 Q2 41.1 Q4 9.93 Q3 6.79
BA.1 MOI=4
Q1 44.8 Q2 50.2 Q4 3.10 Q3 1.91
PDL1
CD80
c
DC2
%PDL1+CD80+
**
**
Medium
R848
0.4
4
B220
0.4
4
Delta
0.4
4
BA.1
d
Monocytes
%PDL1+/CD80+
ns
Medium
R848
0.4
4
B220
0.4
4
Delta
0.4
4
BA.1

Fig. S6

Figure S6 DC2 and monocytes response to Sars-CoV-2 viral variant stimulation.
a. DC2 activation, defined by PDL1 and CD80 expression, stimulated by Medium, R848 and SARS-CoV-2 variants (MOI=0.4 and 4). Representative plots from 1 healthy donor out of 5. **b**. Monocytes $CD88^+$ activation, defined by PDL1 and CD80 expression, stimulated by Medium, R848 and Sars-CoV-2 variants (MOI=0.4 and 4). Representative plots from 1 healthy donor out of 6. **c**. Quantification of $PDL1^+/CD80^+$ DC2 cells. **d.** Quantification of $PDL1^+/CD80^+$ monocytes. Histograms represent means and bars SD of n=6 healthy donors from three independent experiments. *, P < 0.05; **, P < 0.01; Mann–Whitney test.

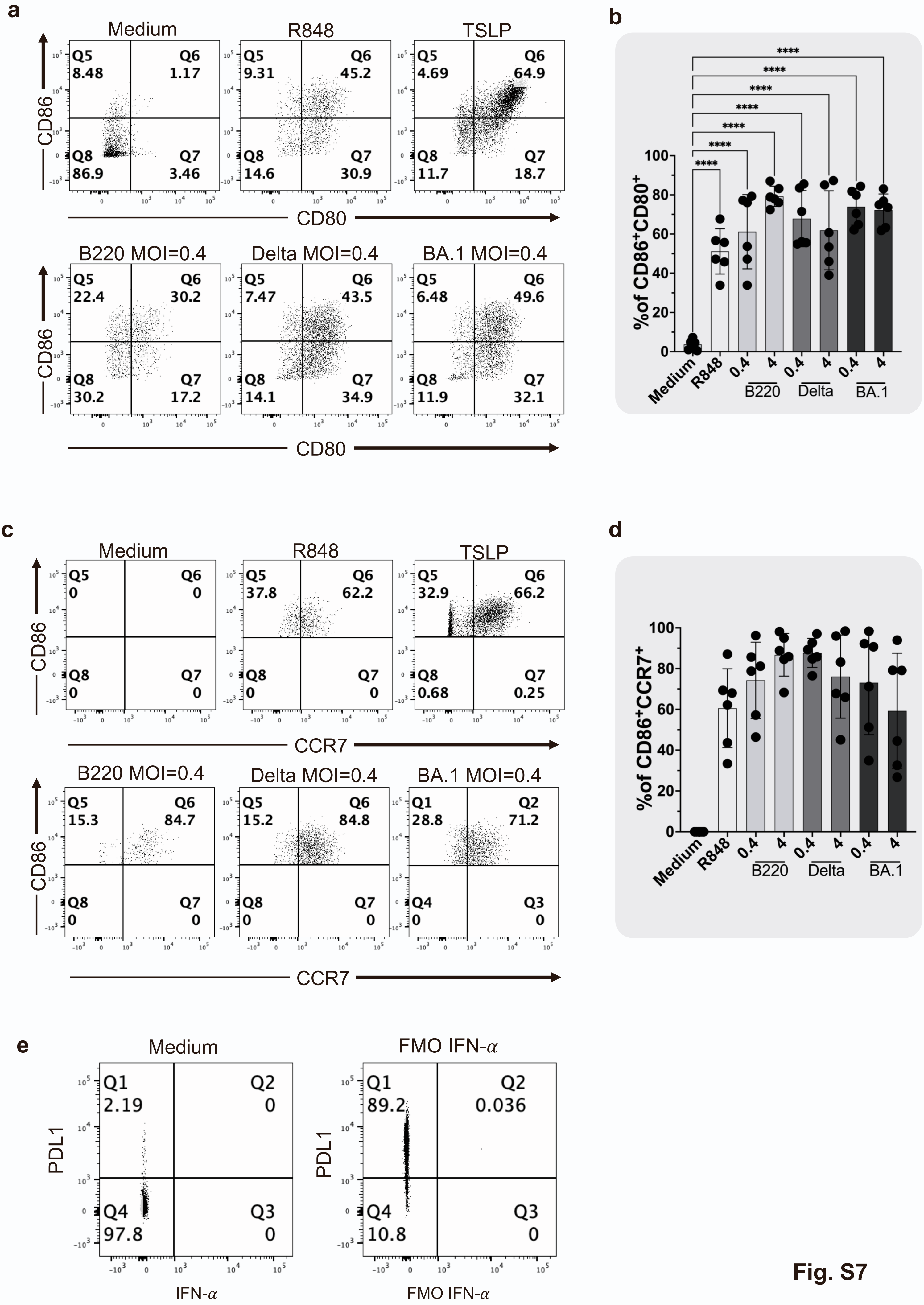
a
Medium
R848
TSLP
Q5 8.48
Q6 1.17
Q8 86.9
Q7 3.46
Q5 9.31
Q6 45.2
Q8 14.6
Q7 30.9
Q5 4.69
Q6 64.9
Q8 11.7
Q7 18.7
CD86
CD80
B220 MOI=0.4
Delta MOI=0.4
BA.1 MOI=0.4
Q5 22.4
Q6 30.2
Q8 30.2
Q7 17.2
Q5 7.47
Q6 43.5
Q8 14.1
Q7 34.9
Q5 6.48
Q6 49.6
Q8 11.9
Q7 32.1
b
****
%of CD86+CD80+
Medium
R848
0.4
4
B220
Delta
BA.1
c
Medium
R848
TSLP
Q5 0
Q6 0
Q8 0
Q7 0
Q5 37.8
Q6 62.2
Q8 0
Q7 0
Q5 32.9
Q6 66.2
Q8 0.68
Q7 0.25
CCR7
B220 MOI=0.4
Delta MOI=0.4
BA.1 MOI=0.4
Q5 15.3
Q6 84.7
Q8 0
Q7 0
Q5 15.2
Q6 84.8
Q8 0
Q7 0
Q1 28.8
Q2 71.2
Q4 0
Q3 0
d
%of CD86+CCR7+
e
Medium
FMO IFN-α
Q1 2.19
Q2 0
Q4 97.8
Q3 0
Q1 89.2
Q2 0.036
Q4 10.8
Q3 0
PDL1
IFN-α
FMO IFN-α

Fig. S7

Figure S7 Expression of co-stimulatory and migration markers (CD80, CD86, CCR7) following SARS-CoV-2 exposure on DC2. IFN-α intracellular controls

a. Representative Flow Cytometry plots of CD80/CD86 expression on DC2 upon SARS-CoV-2 variants stimulation. **b.** Quantification of $CD80^{+}CD86^{+}$ DC2 cells**.** Two way Anova test with Geisser-Greenhouse correction. Bars represent means ± SD. *, P < 0.05; **, P < 0.01 ***, P < 0.001; ****, P < 0.0001. **c.** % of $CD86^{+}CCR7^{+}$ DC2 cells upon Medium, R848 and SARS-CoV-2 variants stimulation at 100 genomes copies/cell. d. Quantification of $CD86^{+}CCR7^{+}$ DC2 cells. e. Representative Flow Cytometry plots of non stimulated pDC IFN-α intracellular production as well as FMO IFN-α control. Histograms represent means and bars SD of n=6 healthy donors from three independent experiments. *, P < 0.05; **, P < 0.01; Mann–Whitney test.

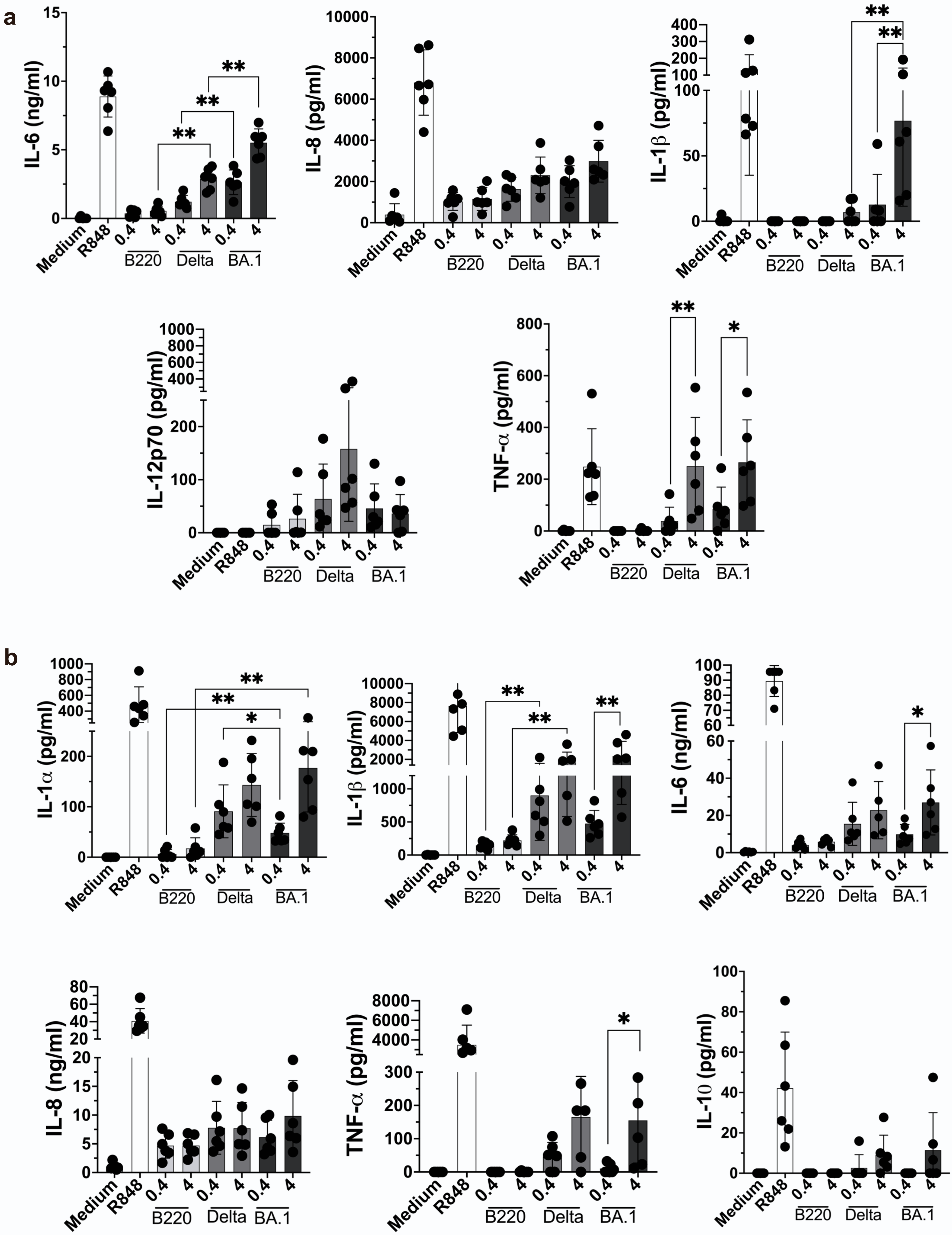
a
IL-6 (ng/ml)
IL-8 (pg/ml)
IL-1β (pg/ml)
IL-12p70 (pg/ml)
TNF-α (pg/ml)
b
IL-1α (pg/ml)
IL-1β (pg/ml)
IL-6 (ng/ml)
IL-8 (ng/ml)
TNF-α (pg/ml)
IL-10 (pg/ml)
Medium
R848
0.4
4
B220
Delta
BA.1
*
**

Fig. S8

Figure S8: SARS-CoV-2 viral variants activates pro-inflammatory cytokine production by DC2 and monocytes. Cytokines production by purified blood DC2 and $CD88^+$ monocytes from healthy donors after 20h of co-culture with medium, R848 and B220, Delta, BA.1 SARS-CoV-2 viral variants at MOI= 0.4 or 4 **a.** Quantification of secreted pro-inflammatory DC2 cytokines after 20 h of culture. **b.** Quantification of secreted proinflammatory cytokines of $CD88^+$ monocytes after 20 h of culture. Histograms represent means and bars SD of n=6 healthy donors from three independent experiments. *, $P < 0.05$; **, $P < 0.01$; Mann–Whitney test.

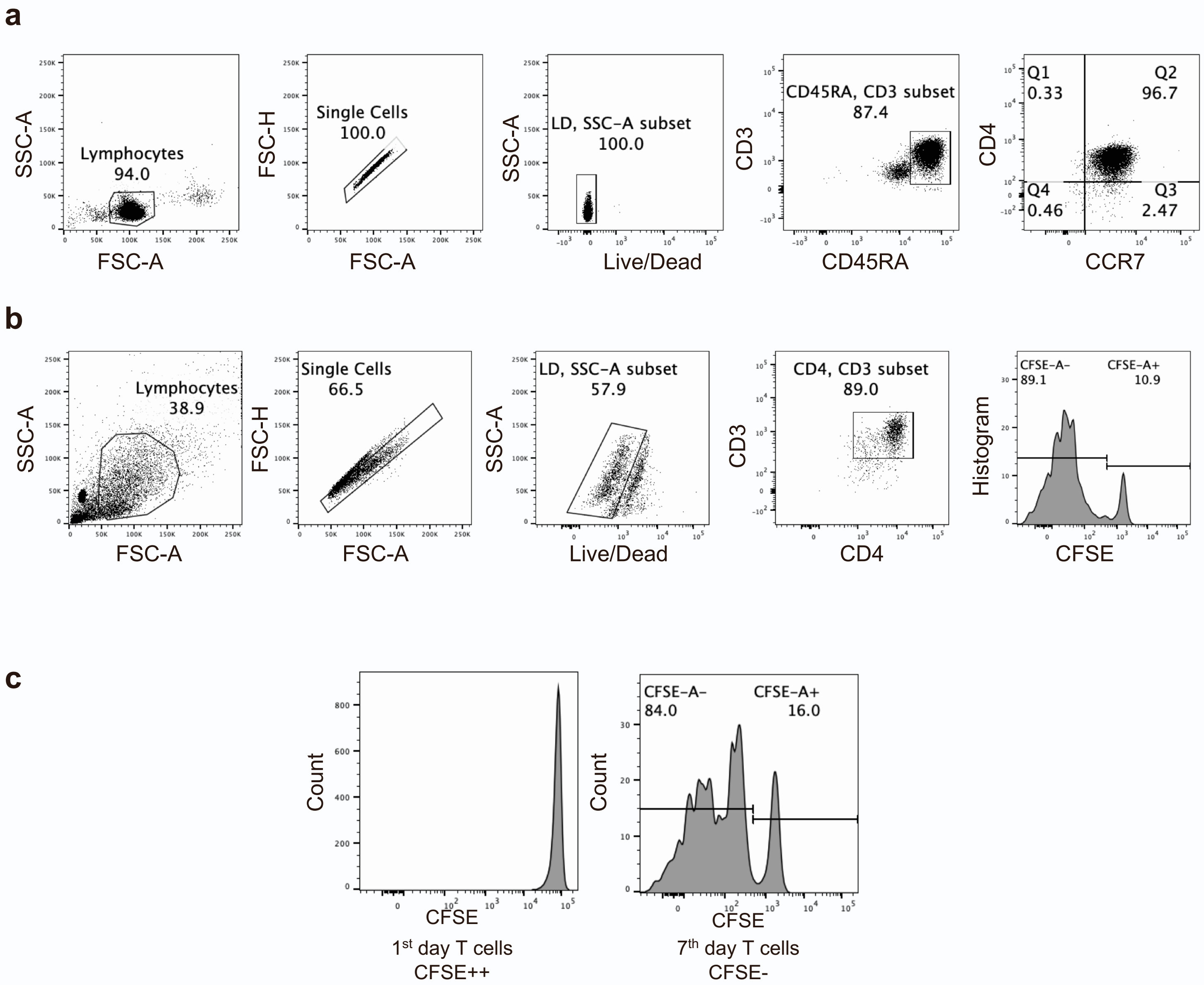
a
Lymphocytes
94.0
SSC-A
FSC-A
Single Cells
100.0
FSC-H
FSC-A
LD, SSC-A subset
100.0
SSC-A
Live/Dead
CD45RA, CD3 subset
87.4
CD3
CD45RA
Q1
0.33
Q2
96.7
Q4
0.46
Q3
2.47
CD4
CCR7
b
Lymphocytes
38.9
SSC-A
FSC-A
Single Cells
66.5
FSC-H
FSC-A
LD, SSC-A subset
57.9
SSC-A
Live/Dead
CD4, CD3 subset
89.0
CD3
CD4
CFSE-A-
89.1
CFSE-A+
10.9
Histogram
CFSE
c
Count
CFSE
1st day T cells
CFSE++
CFSE-A-
84.0
CFSE-A+
16.0
Count
CFSE
7th day T cells
CFSE-

Fig. S9

Figure S9: Gating strategy and purity of T cells after Naive CD4+ T cells isolation StemCell kit. Controls for FN-a intracellular detection.
a. Representative Flow Cytometry plots of isolated from the blood of healthy donors naive CD4+ T cells, defined like CD45RA+CD3+CD4+CCR7+ cells. **b.** Representative Flow Cytometry plots of gating strategy to define T cells phenotype and state of activation after 7 days of co culture and 24h CD3/CD28 dynebeads stimulation on day 6 of the experiment. **c**. Histograms of CFSE-staining, day one of the co culture and day 7.
